# TonEBP inhibits ciliogenesis by controlling aurora kinase A and regulating centriolar satellite integrity

**DOI:** 10.1186/s12964-024-01721-8

**Published:** 2024-07-03

**Authors:** Batchingis Chinbold, Hyug Moo Kwon, Raekil Park

**Affiliations:** 1 Department of Biomedical Science and Engineering, Gwangju Institute of Science and Technology, Gwangju, 61005 Republic of Korea; 2 School of Life Sciences, Ulsan National Institute of Science and Technology, Ulsan, Republic of Korea

**Keywords:** Ciliogenesis, TonEBP, Centriolar satellite, Aurora kinase A, Γ-tubulin

## Abstract

**Background:**

Primary cilia on the surface of eukaryotic cells serve as sensory antennas for the reception and transmission in various cell signaling pathways. They are dynamic organelles that rapidly form during differentiation and cell cycle exit. Defects in these organelles cause a group of wide-ranging disorders called ciliopathies. Tonicity-responsive enhancer-binding protein (TonEBP) is a pleiotropic stress protein that mediates various physiological and pathological cellular responses. TonEBP is well-known for its role in adaptation to a hypertonic environment, to which primary cilia have been reported to contribute. Furthermore, TonEBP is involved in a wide variety of other signaling pathways, such as Sonic Hedgehog and WNT signaling, that promote primary ciliogenesis, suggesting a possible regulatory role. However, the functional relationship between TonEBP and primary ciliary formation remains unclear.

**Methods:**

TonEBP siRNAs and TonEBP-mCherry plasmids were used to examine their effects on cell ciliation rates, assembly and disassembly processes, and regulators. Serum starvation was used as a condition to induce ciliogenesis.

**Results:**

We identified a novel pericentriolar localization for TonEBP. The results showed that TonEBP depletion facilitates the formation of primary cilia, whereas its overexpression results in fewer ciliated cells. Moreover, TonEBP controlled the expression and activity of aurora kinase A, a major negative regulator of ciliogenesis. Additionally, TonEBP overexpression inhibited the loss of CP110 from the mother centrioles during the early stages of primary cilia assembly. Finally, TonEBP regulated the localization of PCM1 and AZI1, which are necessary for primary cilia formation.

**Conclusions:**

This study proposes a novel role for TonEBP as a pericentriolar protein that regulates the integrity of centriolar satellite components. This regulation has shown to have a negative effect on ciliogenesis. Investigations into cilium assembly and disassembly processes suggest that TonEBP acts upstream of the aurora kinase A - histone deacetylase 6 signaling pathway and affects basal body formation to control ciliogenesis. Taken together, our data proposes previously uncharacterized regulation of primary cilia assembly by TonEBP.

**Supplementary Information:**

The online version contains supplementary material available at 10.1186/s12964-024-01721-8.

## Background

Primary cilia are microtubule-based organelles localized on the cell surface that serve as sensors of the extracellular environment and are involved in multiple cell-signaling stimuli during tissue homeostasis and development [1]. Ciliogenesis, generation of primary cilia, is a crucial and complex process involving the trafficking of pre-ciliary vesicles at the centrioles, formation of basal bodies from mother centrioles, and consequent accumulation of ciliary components that allows the axoneme and ciliary vesicle to grow and develop into primary cilia [2]. Moreover, ciliogenesis is tightly regulated by centriolar satellites, a group of mobile membraneless granules that localize and travel around centrioles [3, 4]. Centriolar satellites, which control the integration of necessary ciliary components that form the primary cilium, are essential for efficient ciliogenesis [5–7]. Defects in primary ciliogenesis have been associated with a group of genetic disorders called ciliopathies, which affect different organ systems and often manifest with a wide range of symptoms including developmental delays, renal anomalies, and retinal degeneration [8]. Multitude of different ion channels and receptors that mediate the transduction of cell signaling via Hedgehog signaling, Wnt signaling, neurotransmitters, and growth factors are contained in primary cilia [9–12]. Owing to the importance of ciliated cells, the assembly and disassembly processes of primary cilia are heavily studied [2, 13].

Tonicity-responsive enhancer binding protein (TonEBP), also known as nuclear factor of activated T-cells 5 (NFAT5), a member of the Rel family of transcription factors, was first identified as a key transcription factor activated in response to changes in tonicity [14]. In response to hyperosmolarity, TonEBP translocates to the nucleus and activates the transcription of multiple target genes, further contributing to the accumulation of organic osmolytes that regulate osmotic pressure [15–17]. Interestingly, primary cilia are reported to contribute to the activation of TonEBP in response to hypertonicity [18]. Additionally, numerous studies have shown that TonEBP plays a critical role in a variety of non-tonicity-related cellular processes, such as WNT and Sonic Hedgehog signaling, which in turn promotes primary cilia formation [19–22] Furthermore, TonEBP interacts with other proteins in the cytosol and contributes non-transcriptionally to physiological processes, such as differentiation and autophagy [19, 23]. Significantly, TonEBP has been suggested to co-localize and interact with histone deacetylase 6 (HDAC6), a key mediator of primary cilia disassembly process [24]. Nonetheless, direct evidence for the role of TonEBP in ciliogenesis is obscure.

This study aimed at observing the relationship between TonEBP and ciliogenesis. The results showed a novel negative regulatory relationship between the two. We found that TonEBP localizes near the pericentriolar area to regulate centriolar satellite integrity. Correspondingly, TonEBP silencing increased primary ciliogenesis, whereas TonEBP overexpression suppressed it.

## Methods

### Cell culture

Human telomerase reverse transcriptase-immortalized retinal pigmented epithelial (RPE1) cells were cultured in DMEM/F12 (11,320,033; Gibco-BRL) supplemented with 10% fetal bovine serum (FBS) and 100 IU/ml penicillin (12,140,122; Gibco-BRL) at 37 °C and 5% CO_2_ in a humidified atmosphere. All cultures were confirmed negative for mycoplasma contamination. In all experiments, cells were maintained at approximately 80% confluence to avoid ciliogenesis caused by direct contact inhibition.

### siRNA and plasmid transfection

siRNA transfection was performed using Lipofectamine RNAiMAX (13778075; Invitrogen) according to the manufacturer’s protocol. Cells were fixed at 24–48 h after transfection. siRNA sequences used in this study were; NFAT5 (#1) 5’-GACCAUGGUCCAAAUGCAA-3’ (10725-1, Bioneer) and NFAT5 TonEBP (#2) 5’-CUGUAGUGUUGCAAGUGUU-3’ (10725-3, Bioneer). Plasmid transfection was performed using Lipofectamine 3000 reagent (L3000001, Invitrogen, CA, USA, ) according to the manufacturer’s protocol. Cells were fixed and harvested at 24–48 h after transfection. CMV-NFAT5-mCherry plasmid encoding the full-length version of wild-type human TonEBP with cytomegalovirus promoter was generated by Vector Builder (VB210324-1296qvx).

### Reagents

Following primary antibodies were used in this study: rabbit anti-NFAT5 (PA1-023, Invitrogen), mouse Alexa Fluor 647 anti-gamma (γ) tubulin (ab191114, Abcam), mouse anti-acetylated tubulin (T7451, Sigma-Aldrich), rabbit anti-ARL13b (17711-1-AP, Proteintech), chicken anti-mCherry (TA150127, OriGene), rabbit anti-aurora kinase A (4718, Cell Signaling), rabbit anti-phosphorylated aurora kinase A (2914, Cell Signaling), rabbit anti-Rab11 (71-5300, Invitrogen), rabbit anti-CP110 (ab243696, Abcam), rabbit anti-IFT20 (13615-1-AP, Proteintech), rabbit anti-PCM1 (G2000, Cell Signaling), anti-beta actin HRP (sc-47778, Santa Cruz). HDAC6 inhibitor, Tubastatin A was purchased from APExBIO (A4101). Aurora kinase A primer sequence used in this study is F: 5’-GCAGATTTTGGGTGGTCAGT-3’, R: 5’-TCCGACCTTCAATCATTTCA-3’ (Macrogen).

### Immunofluorescence

Cells grown on coverslips were fixed with methanol at -20 °C for 10 min. Cells were rinsed thrice with PBS (10,010,023, Gibco) and followed by blocking with 3% bovine serum albumin (BSA; 22,070,004, BioWorld) for 1 h at room temperature. Cells were then incubated overnight with primary antibodies in 3% BSA, rinsed thrice with PBS, and labeled with fluorescent Alexa Fluor 488 or Alexa Fluor 568 conjugated secondary antibodies for 1 h. To detect the nuclei, coverslips were mounted with Prolong Gold antifade reagent containing DAPI (P36931, Sigma) and examined under EVOS M7000 Imaging System (#AMF7000, Invitrogen).

### Western blot

Cells in the culture were washed with PBS, harvested, and centrifuged at 5,000 rpm for 5 min at 4 °C. Cell pellets were resuspended in RIPA lysis buffer (20 mM HEPES pH 7.5, 150 mM NaCl, 1% Triton X-100, 1% sodium deoxycholate, 1 mM EDTA), supplemented with protease and phosphatase inhibitors (GenDEPOT, Barker), and centrifuged at 14,000 rpm for 10 min at 4 °C. SDS loading buffer was added to supernatant, which was then denatured at 95 °C for 5 min. Proteins from cell lysates were separated by SDS–PAGE and transferred onto nitrocellulose membranes. Membranes were blocked for 1 h at room temperature with 5% skim milk in Tris-buffered saline containing Tween buffer. Membranes were incubated with primary antibodies overnight at 4 °C, followed by incubation with horseradish peroxidase-conjugated secondary antibodies for 1 h at room temperature, and visualized using Western Blot Detection Kit (LF-QC0103; WestSaveGOLD).

### Statistical analysis

Statistical analysis of the data was performed using student’s t-test. Differences were considered statistically significant at *p* < 0.05.

## Results

### TonEBP is enriched at the pericentriolar area

**Fig. 1:**
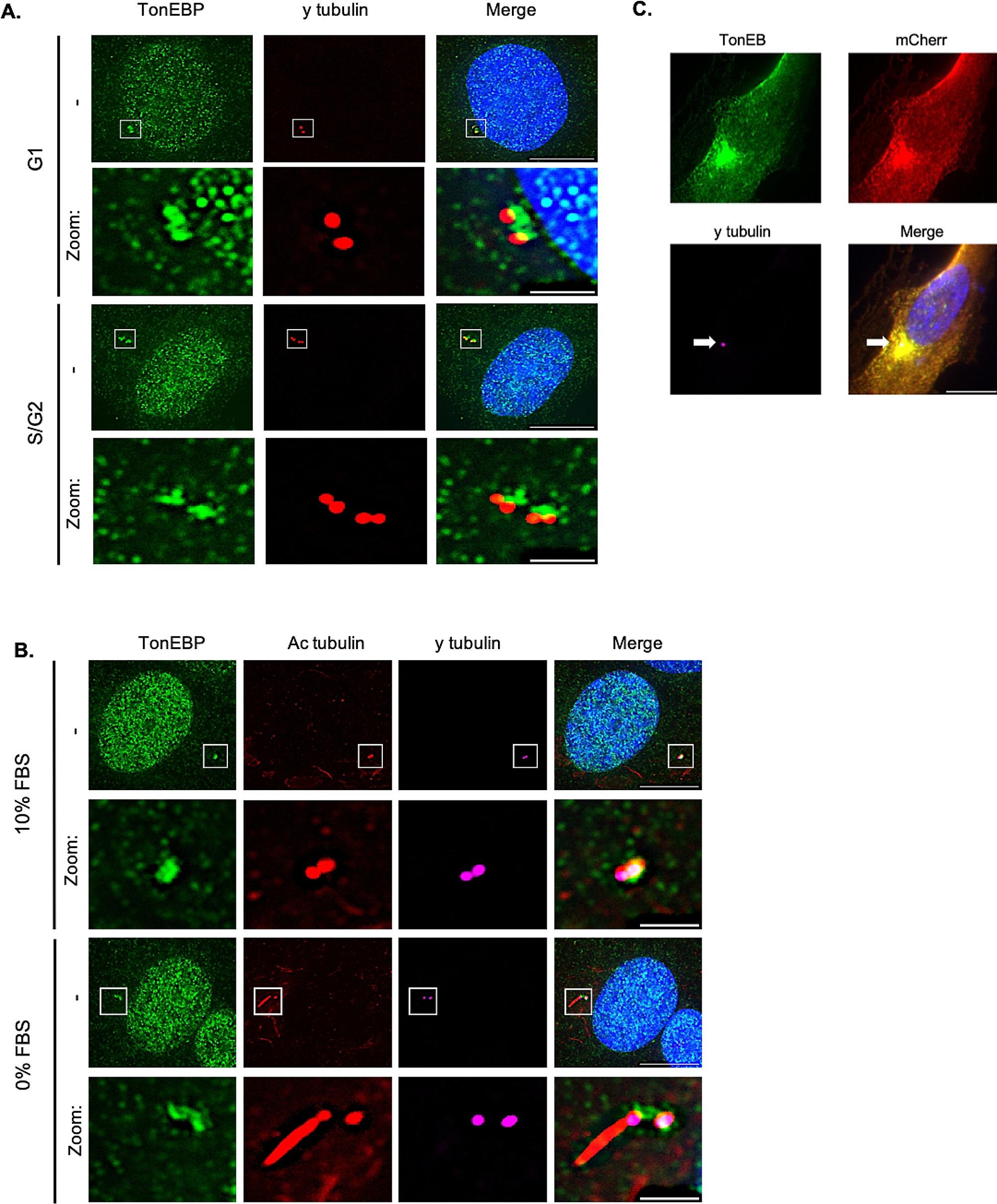
TonEBP is enriched at the pericentriolar area. **a**. RPE1 cells were immunostained for TonEBP (green) and γ-tubulin (red). Scale bar, 10 μm. Zoom scale bar, 2 μm; **b**. Cells were serum staved for 24 h and immunostained for TonEBP (green), acetylated tubulin (red), and γ-tubulin (magenta). Scale bar, 10 μm. Zoom scale bar, 2 μm; **c**. Cells were transfected with TonEBP-mCherry plasmid for 24 h and immunostained for TonEBP (green), mCherry (red) and γ-tubulin (magenta). Scale bar, 10 μm

Because primary cilia are based on mother centrioles [2], we examined TonEBP localization in relation to the centrosome. Towards this end, we used RPE1 cells to stain with antibodies for TonEBP and γ-tubulin. As shown in Fig. 1A, TonEBP was particularly enriched in the nuclei and at the centrosomes, revealing a co-localization to the puncta of γ-tubulin in G1 and S/G2 phases of RPE1 cells. Next, we subjected cells to serum starvation for 24 h to investigate TonEBP localization in ciliated cells. Surprisingly, TonEBP signals remained in both the daughter and mother centrioles of ciliated cells (Fig. 1B). To provide further evidence to support these findings, we overexpressed TonEBP-mCherry in RPE1 cells. Consistently with γ-tubulin localization, TonEBP transfected cells showed accumulation of TonEBP at the centrioles and pericentriolar region (Fig. 1C).

TonEBP responds to hypertonic stress, increasing its expression, and translocates into the nuclei [14]. Accordingly, we examined TonEBP expression and localization during this stimulus. Interestingly, the pericentriolar localization of TonEBP persisted, whereas nuclear TonEBP signals showed higher accumulation with increased intensity of protein expression (Supp. Figure 1A, B.). Taken together, our data implied that TonEBP is a novel and distinguished pericentriolar protein.

### Depletion of TonEBP increases the percentage of ciliated cells in a dose-dependent manner

**Fig. 2:**
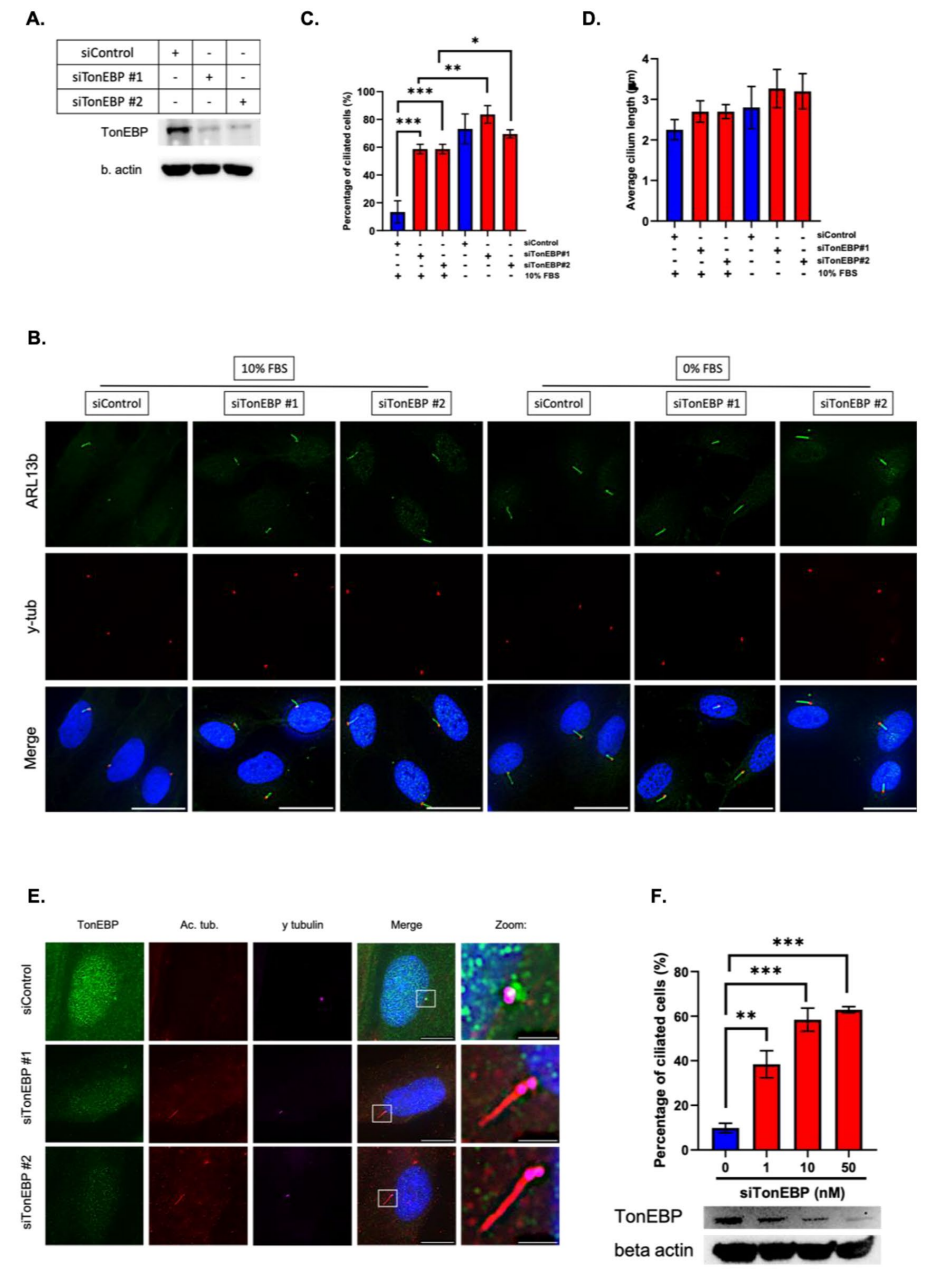
Depletion of TonEBP increases the percentage of ciliated cells in a dose-dependent manner. **a**. RPE1 cells were transfected with TonEBP siRNA for 24 h and used to measure protein expression by Western blot; **b**. Cells were transfected with siRNAs against TonEBP for 24 h, followed by 24 h of serum starvation, and immunostained for ARL13b (green) and γ-tubulin (red). Scale bar, 25 μm; **c**. Quantification of ciliated cell percentage as shown in (B). Data is represented as mean ± SD (*n* = 3 experiments). Two hundred cells were scored per condition per experiment; *P < 0.05, **P < 0.01, ***P < 0.001, Student’s t-test; **d**. Quantification of average primary cilia length as shown in (B). Data is represented as mean ± SD (*n* = 3 experiments). Two hundred cells were scored per condition per experiment; NS > 0.05, Student’s t-test; **e**. Cells were transfected with siRNAs against TonEBP for 24 h, followed by 24 h of serum starvation, and immunostained for TonEBP (green), acetylated tubulin (red), and γ-tubulin (magenta). Scale bar, 10 μm. Zoom scale bar, 2 μm; **f**. Cells were transfected with different concentrations of TonEBP siRNA for 24 h and subjected to Western blot. They were used to quantify the percentage of ciliated cells. Data is represented as mean ± SD (*n* = 3 experiments). Two hundred cells were scored per condition per experiment; **P < 0.01, ***P < 0.001, Student’s t-test

Based on previous findings (Fig. 1), we investigated the role of TonEBP in primary cilia formation. To avoid the off-target effects of siRNA, we used two TonEBP siRNAs, both of which showed high knockdown efficiency (Fig. 2A). Next, we silenced TonEBP for 24 h, followed by serum starvation for 24 h and examined the presence of ciliated cells. Primary cilia were observed in TonEBP-depleted cells under both serum-fed and starved conditions, whereas the control groups presented primary cilia only under serum starvation (Fig. 2B). Notably, the percentage of ciliated cells in TonEBP depletion alone was lower than that in the absence of serum, indicating that certain factors of ciliogenesis are induced by the absence of serum and are not controlled by TonEBP (Fig. 2C). Furthermore, the length of primary cilia in TonEBP depletion conditions was comparable to that in serum-starved conditions, suggesting that TonEBP mainly affects the initiation of ciliogenesis rather than its progression (Fig. 2D). Accordingly, TonEBP depletion-induced ciliated cells displayed an absence of TonEBP signals from the centrioles (Fig. 2E). Finally, we examined the dose-dependent effects of TonEBP depletion. As shown in Fig. 2F, the number of ciliated cells paralleled TonEBP protein levels. Collectively, our data proposed a novel role for TonEBP as a ciliogenesis suppressor in RPE1 cells.

### Overexpression of TonEBP markedly suppresses ciliogenesis

**Fig. 3:**
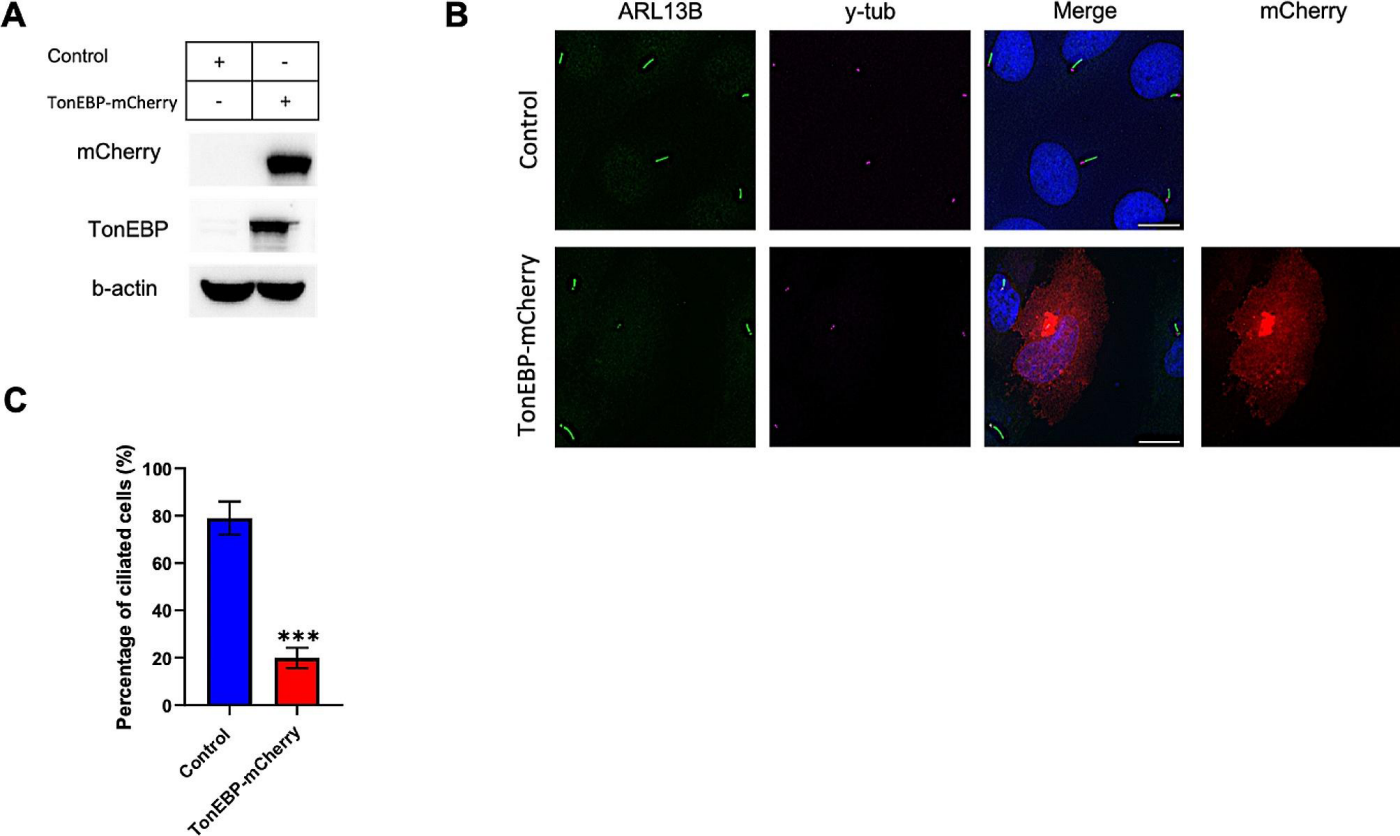
Overexpression of TonEBP markedly suppresses ciliogenesis. **a**. RPE1 cells were transfected with TonEBP-mCherry plasmid for 24 h and then subjected to Western blot; **b**. Cells were transfected with TonEBP-mCherry plasmid for 24 h, followed by serum starvation, and immunostained for ARL13b (green), mCherry (red), and γ-tubulin (magenta). Scale bar, 10 μm; **c**. Quantification of ciliated cell percentage as shown in (B). Data is represented as mean ± SD (*n* = 3 experiments). One hundred fifty cells were scored per condition per experiment; ***P < 0.001, Student’s t-test

Because TonEBP depletion promoted ciliogenesis, we speculated that TonEBP overexpression could inhibit the formation of primary cilia. Therefore, we used TonEBP-mCherry plasmid to examine its effect on ciliation rate. After successful transfection with TonEBP-mCherry (Fig. 3A), cells were further incubated for 24 h, followed by 24 h of serum starvation. Consequently, TonEBP overexpression significantly blocked primary ciliogenesis compared to non-transfected cells under serum-starved conditions (Fig. 3B, C). These results confirmed the inhibitory role of TonEBP in primary ciliary formation.

### TonEBP regulates the expression and activity of aurora A kinase

**Fig. 4:**
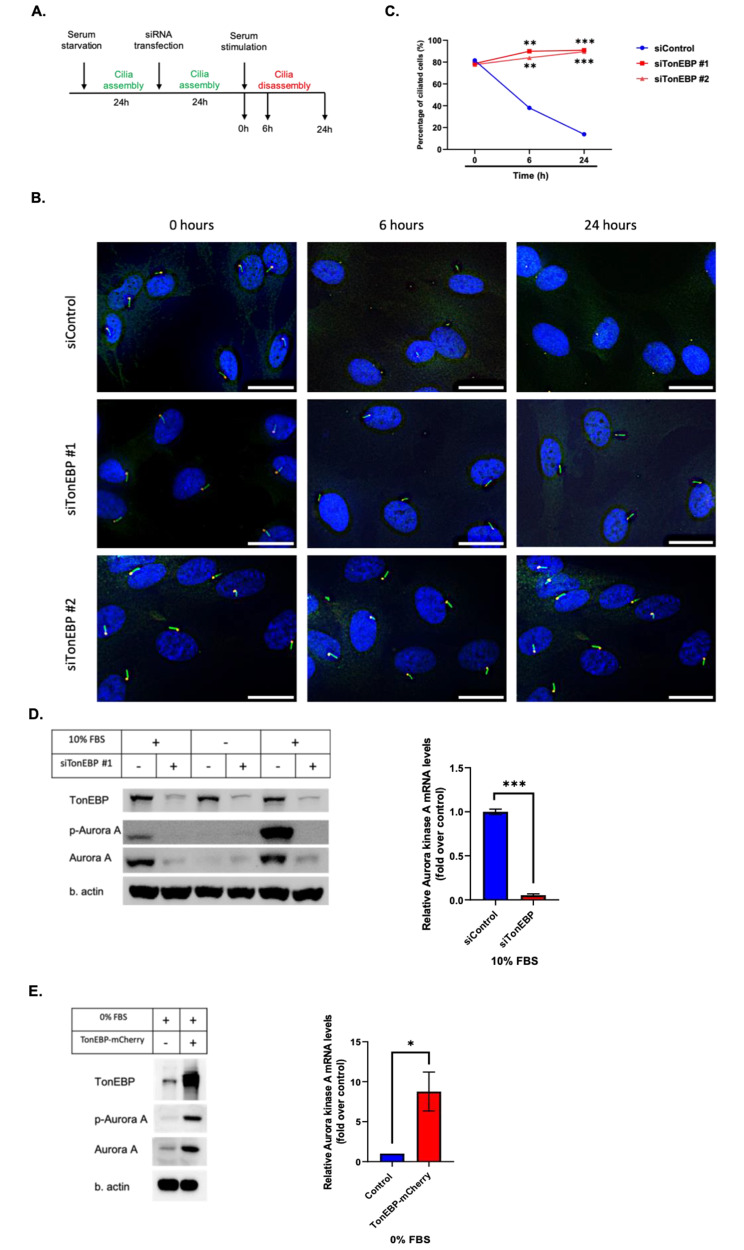
TonEBP regulates the expression and activity of aurora A kinase. **a**. RPE1 cells were serum-starved for 24 h, transfected with TonEBP siRNA for 24 h, followed by serum stimulation, and harvested at different time points, as indicated; **b**. Cells were transfected with siRNAs against TonEBP for 24 h, followed by 24 h of serum starvation, and immunostained for ARL13b (green) and γ-tubulin (red). Scale bar, 25 μm; **c**. Quantification of ciliated cell percentage as shown in (B). Data is represented as mean ± SD (*n* = 3 experiments). Two hundred cells were scored per condition per experiment; **P < 0.01, ***P < 0.001, Student’s t-test; **d**. Cells were transfected with TonEBP siRNA for 24 h, serum-starved, followed by serum restimulation. Then, cells were harvested at different time points and subjected to Western blot and qPCR. qPCR data is represented as mean ± SD (*n* = 3 experiments). ***P < 0.001, Student’s t-test; **e**. Cells were transfected with TonEBP-mCherry plasmid for 24 h, followed by serum starvation, and subjected to Western blot and qPCR. qPCR data is represented as mean ± SD (*n* = 3 experiments). *P < 0.05, Student’s t-test

Primary cilia dynamics are dependent on primary cilia assembly and disassembly factors [2, 13]. Although serum starvation is widely used to study cilium formation, serum restimulation reflects primary cilium disassembly [13, 25]. Given its inhibitory role in ciliogenesis, we examined whether TonEBP affects primary cilium resorption. As shown in Fig. 4A, we first serum-starved RPE1 cells, followed by TonEBP knockdown for 24 h, and then re-stimulated the cells with serum. While control cells showed a dramatic decrease in the number of ciliated cells as early as 6 h of serum re-stimulation and displayed minimal ciliation levels at 24 h, depletion of TonEBP with two different siRNA constructs suppressed primary cilia disassembly, even after 24 h of serum stimulation (Fig. 4B, C). These observations prompted us to investigate whether TonEBP plays a role in primary cilium disassembly. Aurora kinase A is a major mediator of the primary cilium disassembly process and the main negative regulator of ciliogenesis [26]. Therefore, we examined the relationship between TonEBP and aurora kinase A. First, we depleted TonEBP under serum-fed, starved, and restimulated conditions to examine the aurora kinase A levels. Both total and phosphorylated forms of aurora kinase A disappeared in serum-starved conditions and were rescued only in the presence of serum, whereas in TonEBP-silenced samples, aurora kinase A was completely abolished regardless of serum content. Consequently, depletion of TonEBP showed significant decrease in aurora kinase A mRNA (Fig. 4D). Next, we overexpressed TonEBP in RPE1 cells, followed by serum starvation for 24 h, and analyzed the expression of aurora kinase A. As expected, exogenous TonEBP increased the expression of aurora kinase A, even under serum-starved conditions (Fig. 4E). These results suggested that TonEBP controls the abundance and activity of aurora kinase A.

Next, we inhibited the catalytic activity of aurora kinase A using the potent inhibitor, Alisertib. First, we confirmed the effects of Alisertib on aurora kinase A activity. There was a marked decrease in the level of phosphorylated aurora kinase A in RPE1 cells (Supp. Figure 2A). However, Alisertib failed to rescue ciliogenesis in TonEBP-mCherry-overexpressing cells (Supp. Figure 2B). A recent study reported the co-localization of TonEBP and HDAC6, a downstream effector of aurora kinase A, which deacetylates and destabilizes the ciliary axoneme to promote its disassembly [24, 27]. Hence, we speculated that TonEBP directly activates HDAC6. Therefore, we used the HDAC6 inhibitor, Tubastatin A, which efficiently increased acetylated tubulin levels (Supp. Figure 2C). However, treatment with Tubastatin A did not rescue the primary cilia in TonEBP-mCherry-transfected cells.

Overall, these results suggested that aurora A-HDAC6 pathway is not the sole mediator of TonEBP overexpression-induced suppression of ciliogenesis.

### Overexpression of TonEBP inhibits CP110 degradation from the mother centrioles

**Fig. 5:**
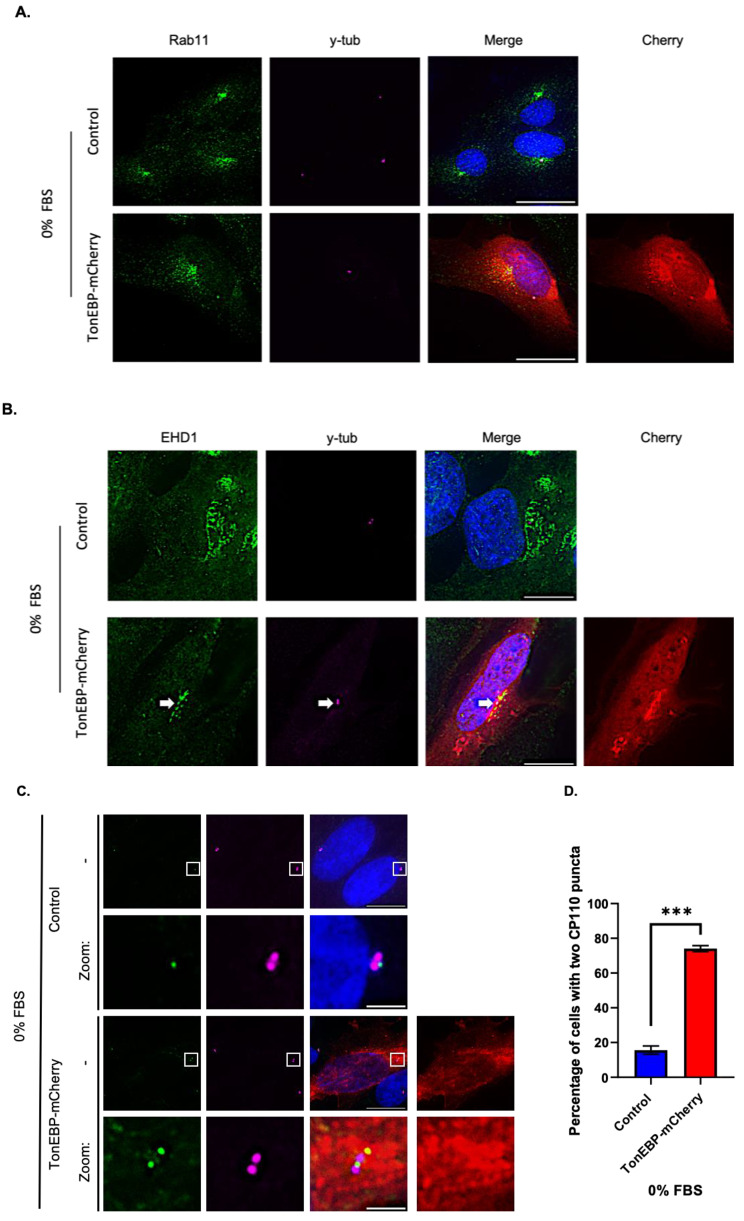
Overexpression of TonEBP inhibits the CP110 removal from mother centrioles to suppress ciliogenesis. **a**. Cells were transfected with TonEBP-mCherry for 24 h, followed by serum starvation 24 h, and stained with antibodies against Rab11 (green), mCherry (red) and γ-tubulin (magenta). Scale bar, 25 μm; **b**. Cells were transfected with TonEBP-mCherry for 24 h, followed by serum starvation 24 h, and stained with antibodies against EHD1 (green), mCherry (red) and γ-tubulin (magenta). Scale bar, 25 μm. Arrows indicate colocalization of EHD1 and y-tubulin; **c**. Cells were transfected with TonEBP-mCherry for 24 h, followed by serum starvation 24 h, and stained with antibodies against CP110 (green), mCherry (red) and γ-tubulin (magenta). Scale bar, 10 μm. Zoom scale bar, 2 μm; **d**. Quantification of the percentage of cells with two CP110 puncta at the centrioles shown in (C). Data is represented as mean ± SD (*n* = 3 experiments). One hundred fifty cells were scored per condition per experiment; ***P < 0.001, Student’s t-test

Because the inhibition of primary cilium disassembly process alone did not rescue ciliogenesis, we examined the process of primary cilium formation. We used Rab11 and EHD1 as markers for pre-ciliary vesicles and examined their trafficking to mother centrioles [28, 29]. As shown in Fig. 5A and B, TonEBP-transfected cells showed accumulation of both Rab11 and EHD1 signals at the centrioles, suggesting that the activation and trafficking of pre-ciliary vesicles were not affected by TonEBP. Next, we examined the formation of basal bodies, a critical early control mechanism for the initiation of ciliogenesis, in which CP110 is removed from mother centrioles to promote ciliary axoneme formation [30, 31]. Interestingly, only around 20% of TonEBP-mCherry-transfected cells displayed the absence of CP110 signals from the mother centrioles, indicating that basal body formation was suppressed by TonEBP transfection (Fig. 5C, D). These results indicated that TonEBP inhibits the formation of basal bodies and thereby ciliogenesis.

### TonEBP regulates PCM1 and AZI1 localization

**Fig. 6:**
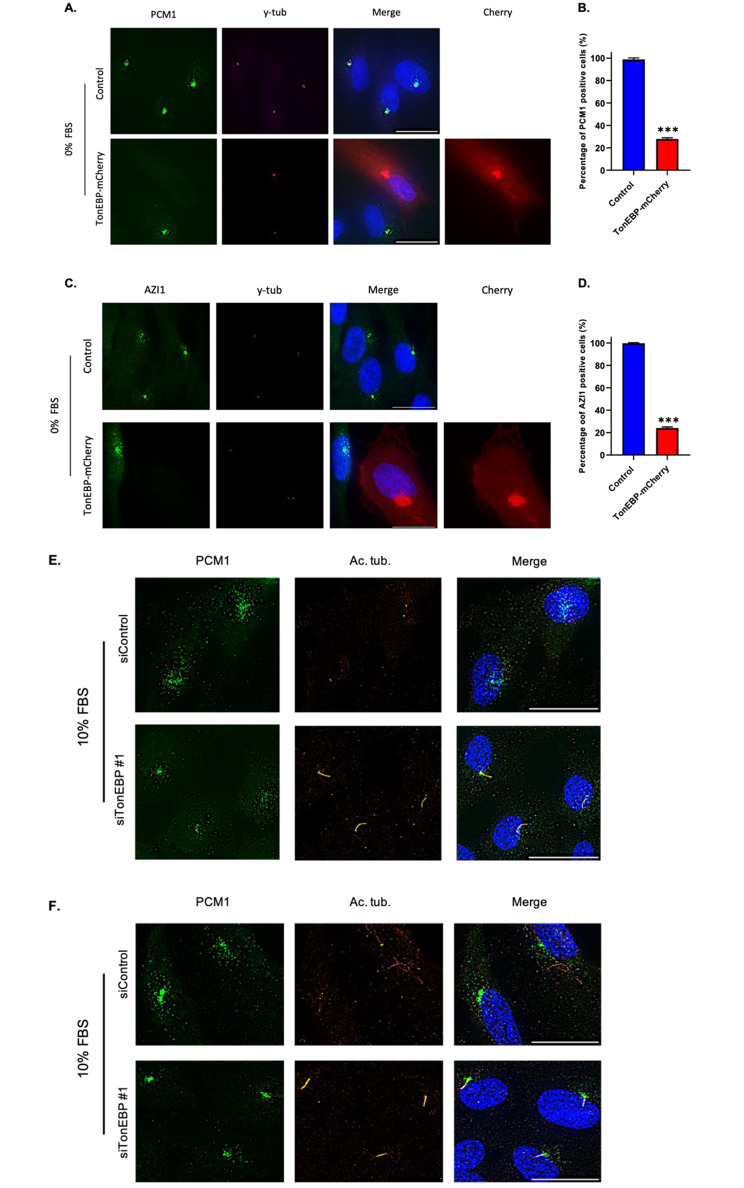
TonEBP regulates PCM1 and AZI1 localization. **a**. RPE1 cells were transfected with TonEBP-mCherry for 24 h, followed by serum starvation 24 h, and immunostained for PCM1 (green), mCherry (red) and γ-tubulin (magenta). Scale bar, 20 μm; **b**. Quantification of the percentage of PCM1 positive cells as shown in (A). Data is represented as mean ± SD (*n* = 3 experiments). Two hundred cells were scored per condition per experiment; ***P < 0.001, Student’s t-test; **c**. Cells were transfected with TonEBP-mCherry for 24 h, followed by serum starvation 24 h, and immunostained for AZI1 (green), mCherry (red) and γ-tubulin (magenta). Scale bar, 20 μm; **d**. Quantification of the percentage of AZI1 positive cells as shown in (A). Data is represented as mean ± SD (*n* = 3 experiments). Two hundred were scored per condition per experiment; ***P < 0.001, Student’s t-test; **e**. Cells were transfected with TonEBP siRNA for 24 h and immunostained for PCM1 (green) and γ-tubulin (red). Scale bar, 20 μm; **f**. Cells were transfected with TonEBP siRNA for 24 h and immunostained for AZI1 (green) and γ-tubulin (red). Scale bar, 20 μm

Recent studies revealed that CP110 loss from the mother centrioles depends on the integrity of centriolar satellites [7, 32, 33]. Because it is considered an essential component of centriolar satellites, we analyzed PCM1 localization in TonEBP-mCherry-transfected cells [5]. Interestingly, cells exhibited minimal PCM1 signaling in the pericentriolar region (Fig. 6A, B). Moreover, AZI1, an additional core member of centriolar satellites, was abolished in TonEBP-overexpressed cells (Fig. 6C, D) [34, 35]. Next, we silenced TonEBP to examine the distribution of PCM1 and AZI1 throughout cells. Accordingly, TonEBP depletion resulted in the accumulation of PCM1 and AZI1 signals in the pericentriolar area, whereas control cells displayed dispersed puncta of these proteins (Fig. 6E, F). These results suggested that TonEBP inhibits ciliogenesis by regulating centriolar satellite integrity.

## Discussion

In this study, we provided evidence for a previously unidentified role of TonEBP in primary ciliogenesis. This was based on the following results. First, TonEBP is enriched in the pericentriolar area. Second, the depletion of TonEBP resulted in robust ciliation, whereas its overexpression inhibited the formation of primary cilia. Third, TonEBP was revealed as an upstream signal of aurora kinase A. Fourth, TonEBP overexpression inhibited the formation of basal bodies during ciliogenesis. Lastly, TonEBP was involved in centriolar satellite organization. Taken together, our results suggest that TonEBP regulates centriolar satellite integrity to control primary cilia assembly. These findings suggested that TonEBP functions in the pericentriolar area to regulate the abundance of centriolar satellite components that constrain the formation of primary cilia.

TonEBP is a dynamic transcription factor that localizes to both the nucleus and cytoplasm. Under relevant stimuli, such as hypertonicity, TonEBP is rapidly trafficked to the nucleus. Therefore, the functional aspect of TonEBP is often studied as a transcription factor in context of investigating its target genes [36]. However, only few publications shed a light on the cytoplasmic role of TonEBP, leaving the subject largely unknown [23, 24]. The cytoplasmic distribution of TonEBP is thought to be largely diffused and pancytosolic [24, 37]. In this study, we observed a significant presence of TonEBP in the nucleus and discovered a compelling accumulation of TonEBP in the pericentriolar region. Interestingly, the persistent pericentriolar localization of TonEBP during serum starvation and hypertonic stress, suggests an independent role of TonEBP as a notable protein of pericentriolar material.

Primary cilia are dynamic organelles that assemble on mother centrioles and basal bodies, and are intricately regulated by pericentriolar material [5–7]. Considering TonEBP as a potential pericentriolar component, we speculated that it is involved in process of ciliogenesis. Surprisingly, TonEBP depletion increased the rate of ciliation under serum-fed conditions. This suggests that TonEBP negatively regulates ciliogenesis. Notably, the combination of serum starvation and TonEBP knockdown resulted in higher levels of ciliation than that in TonEBP knockdown alone, indicating that TonEBP does not exclusively govern ciliogenesis. However, its overexpression dramatically decreased the number of ciliated cells, confirming its suppressive role.

Aurora kinase A is a major mediator of the negative regulation of primary cilia, where it activates histone deacetylase 6 (HDAC6), which destabilizes axonemal microtubules to disassemble the organelle [13, 26, 27]. Furthermore, TonEBP has been proposed to co-localize with HDAC6, suggesting possible functional interactions [24]. TonEBP depletion caused a rapid decrease in aurora kinase A levels, which contributed to an increase in the number of ciliated cells. Moreover, TonEBP-silenced cells retained the abolished levels of aurora kinase A, even after serum re-stimulation, suggesting that TonEBP is required for primary cilium disassembly. TonEBP overexpression consistently elevated the expression and activity of aurora kinase A, suggesting upstream regulation of the latter. However, inhibition of aurora kinase A and HDAC6 did not rescue ciliogenesis in TonEBP-transfected cells, suggesting that the inhibitory effect of TonEBP on primary cilia is not limited to aurora kinase A and HDAC6. Similarly to TonEBP, recent publications have shown that trichoplein inhibits ciliogenesis independent of aurora kinase A, despite its ability to modulate the expression and activity of the latter [34]. Additionally, aurora kinase A localizes to the centrioles in proliferating cells and disappears during ciliogenesis [34, 35]. Unfortunately, we couldn’t detect any aurora kinase A signal using immunofluorescence experiments. Nevertheless, given the novel pericentriolar localization of TonEBP and its upstream regulation, it is plausible that TonEBP is required for the localization of aurora kinase A.

Examination of primary cilia assembly processes revealed that TonEBP overexpression did not affect Rab11 or EHD1 localization to the mother centrioles, suggesting that the trafficking of pre-ciliary vesicles was not affected by TonEBP. Next, EHD1 fuses together pre-ciliary vesicles and assemble into ciliary vesicle, which stimulates the formation of basal bodies [2, 30]. The generation of basal bodies involves the degradation and removal of CP110 protein from the mother centriole to allow nucleation and extension of the ciliary axoneme [2, 31, 32, 38]. Removal of CP110 is a key and decisive event that promotes the formation of primary cilia, and is considered a major negative regulator of ciliogenesis [2, 31]. Interestingly, TonEBP transfection inhibited CP110 loss even with EHD1 positive mother centrioles. These results were similar to recent report, where basal body formation was not dependent on pre-ciliary/ciliary vesicle [39, 40].

Several studies reported that centriolar satellites regulate the formation of basal bodies [7, 32, 33]. Interestingly, under serum-starved conditions, TonEBP was observed at the centrosomes, suggesting a reorganizing role in the pericentriolar region and ciliogenesis. Accordingly, TonEBP-transfected cells displayed a distinct lack of PCM1 signal at the pericentriolar area. PCM1, as a fundamental and core member of centriolar satellites, regulates the abundance of other centriolar satellite components, such as AZI1, and is thought to be required for ciliogenesis [5, 7, 41–43]. Correspondingly, TonEBP overexpression abolished the localization of AZI1. Moreover, TonEBP depletion resulted in increased aggregation of PCM1 and AZI1 in the pericentriolar area, providing additional functional evidence for the role of TonEBP as a pericentriolar protein. Centriolar satellites have been notably proposed to regulate the abundance and activity of aurora kinase A at the centrioles [35]. This may explain the relationship between TonEBP and aurora kinase A. The integrity of centriolar satellites is dependent on multitude of factors such as intact microtubule network, protein turnover/trafficking, cellular stresses and posttranslational modification of PCM1 [4]. The centriolar satellite abundance is also dependent on mother centriole itself, as depletion of various distal appendage proteins abolished PCM1 niche at the pericentriolar area [44]. Moreover, DISC1 and BBS4 have been shown to be required to PCM1 localization as well and are synergistically associated with mental pathologies, such as schizophrenia [45]. Given this vast network of governance, additional investigations are required to elucidate the precise impact of TonEBP on PCM1 distribution.

## Conclusion

**Fig. 7:**
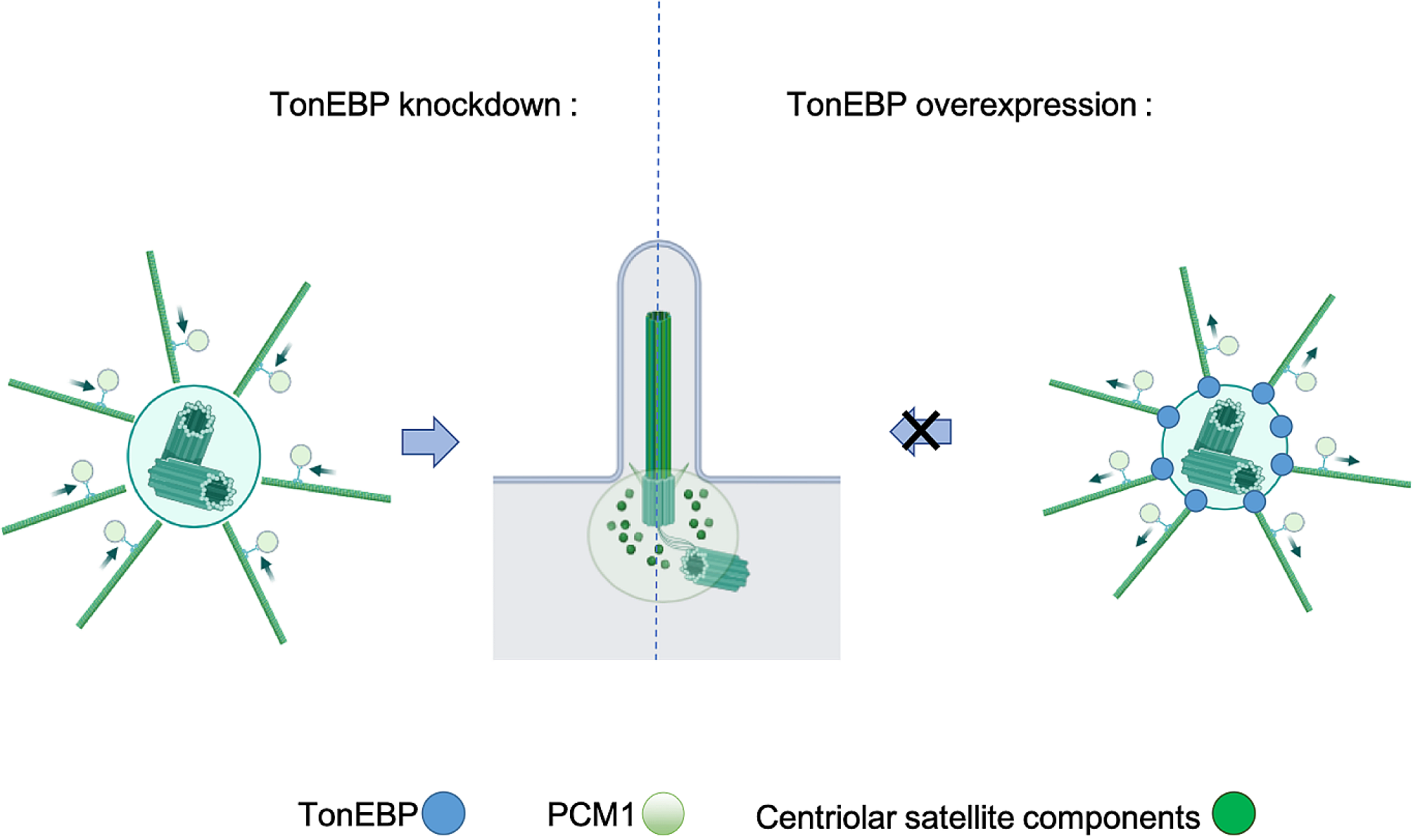
A conceptual model proposing the regulatory influence of TonEBP on ciliogenesis

Overall, our observations provide a novel functional link between TonEBP and ciliogenesis, revealing a new physiological function of TonEBP. Pericentriolar localization of TonEBP contributes to the centriolar satellite organization. Disruption of centriolar satellite integrity by TonEBP overexpression prevents the removal of CP110 from mother centrioles and the formation of basal bodies to promote ciliogenesis. Additionally, TonEBP is an upstream regulator of aurora kinase A expression and activity. Therefore, these results indicate that pericentriolar TonEBP controls aurora kinase A and regulates the integrity of centriolar satellites at the early stages of ciliogenesis (Fig. 7). Further research in required to unravel the exact mechanism through which TonEBP contributes to centriolar satellites organization and ciliogenesis.

### Electronic supplementary material

Below is the link to the electronic supplementary material.

Supplementary Material 1

Supplementary Material 2

## Data Availability

No datasets were generated or analysed during the current study.

## References

[1] Gerdes JM, Davis EE, Katsanis N. The vertebrate primary cilium in development, homeostasis, and disease. Cell. 2009;137(1):32–45. 10.1016/j.cell.2009.03.023. 10.1016/j.cell.2009.03.023 PMC3016012 19345185

[2] Wang L, Dynlacht BD. The regulation of cilium assembly and disassembly in development and disease. Development. 2018;145(18):dev151407. 10.1242/dev.151407. 10.1242/dev.151407 PMC6176931 30224385

[3] Prosser SL, Pelletier L. Centriolar satellite biogenesis and function in vertebrate cells. J Cell Sci. 2020;133(1):jcs239566. 10.1242/jcs.239566. 10.1242/jcs.239566 31896603

[4] Hori A, Toda T (2017). Regulation of centriolar satellite integrity and its physiology. Cell Mol Life Sci.

[5] Odabasi E, Gul S, Kavakli IH, Firat-Karalar EN (2019). Centriolar satellites are required for efficient ciliogenesis and ciliary content regulation. EMBO Rep.

[6] Wang L, Lee K, Malonis R, Sanchez I, Dynlacht BD. Tethering of an E3 ligase by PCM1 regulates the abundance of centrosomal KIAA0586/Talpid3 and promotes ciliogenesis. Elife. 2016;5:e12950. 10.7554/eLife.12950. 10.7554/eLife.12950 PMC4858382 27146717

[7] Hall EA, Kumar D, Prosser SL, et al. Centriolar satellites expedite mother centriole remodeling to promote ciliogenesis. Elife. 2023;12:e79299. 10.7554/eLife.79299. 10.7554/eLife.79299 PMC9998092 36790165

[8] Hildebrandt F, Benzing T, Katsanis N (2011). Ciliopathies. N Engl J Med.

[9] McMahon AP, Ingham PW, Tabin CJ. Developmental roles and clinical significance of hedgehog signaling. Curr Top Dev Biol. 2003;53:1–114. 10.1016/s0070-2153(03)53002-2. 10.1016/s0070-2153(03)53002-2 12509125

[10] He X (2008). Cilia put a brake on wnt signalling. Nat Cell Biol.

[11] Stubbs T, Koemeter-Cox A, Bingman JI et al. Disruption of dopamine receptor 1 localization to primary cilia impairs signaling in striatal neurons [published online ahead of print, 2022 Jul 25]. J Neurosci. 2022;42(35):6692–705. 10.1523/JNEUROSCI.0497-22.2022. 10.1523/JNEUROSCI.0497-22.2022 PMC9436016 35882560

[12] Chinipardaz Z, Liu M, Graves DT, Yang S. Role of primary cilia in bone and cartilage. J Dent Res. 2022;101(3):253–60. 10.1177/00220345211046606. 10.1177/00220345211046606 PMC8866845 34743626

[13] Patel MM, Tsiokas L. Insights into the regulation of Ciliary Disassembly. Cells. 2021;10(11):2977. 10.3390/cells10112977. 10.3390/cells10112977 PMC8616418 34831200

[14] Miyakawa H, Woo SK, Dahl SC, Handler JS, Kwon HM (1999). Tonicity-responsive enhancer binding protein, a rel-like protein that stimulates transcription in response to hypertonicity. Proc Natl Acad Sci U S A.

[15] Rim JS, Atta MG, Dahl SC, Berry GT, Handler JS, Kwon HM. Transcription of the sodium/myo-inositol cotransporter gene is regulated by multiple tonicity-responsive enhancers spread over 50 kilobase pairs in the 5’-flanking region. J Biol Chem. 1998;273(32):20615–21. 10.1074/jbc.273.32.20615. 10.1074/jbc.273.32.20615 PMC2365891 9685419

[16] Miyakawa H, Woo SK, Chen CP, Dahl SC, Handler JS, Kwon HM. Cis- and trans-acting factors regulating transcription of the BGT1 gene in response to hypertonicity. Am J Physiol. 1998;274(4):F753–61. 10.1152/ajprenal.1998.274.4.F753. 10.1152/ajprenal.1998.274.4.F753 9575900

[17] Hasler U, Jeon US, Kim JA (2006). Tonicity-responsive enhancer binding protein is an essential regulator of aquaporin-2 expression in renal collecting duct principal cells. J Am Soc Nephrol.

[18] Siroky BJ, Kleene NK, Kleene SJ, et al. Primary cilia regulate the osmotic stress response of renal epithelial cells through TRPM3. Am J Physiol Ren Physiol. 2017;312(4):F791–805. 10.1152/ajprenal.00465.2015. 10.1152/ajprenal.00465.2015 PMC5407065 28122715

[19] Wang Q, Zhou Y, Rychahou P, Liu C, Weiss HL, Evers BM. NFAT5 represses canonical wnt signaling via inhibition of β-catenin acetylation and participates in regulating intestinal cell differentiation. Cell Death Dis. 2013;4(6):e671. 10.1038/cddis.2013.202. 10.1038/cddis.2013.202 PMC3702276 23764852

[20] Kyun ML, Kim SO, Lee HG et al. Wnt3a stimulation promotes primary ciliogenesis through β-Catenin Phosphorylation-Induced reorganization of Centriolar satellites. Cell Rep. 2020;30(5):1447–e625. 10.1016/j.celrep.2020.01.019. 10.1016/j.celrep.2020.01.019 32023461

[21] Tessier S, Madhu V, Johnson ZI, Shapiro IM, Risbud MV (2019). NFAT5/TonEBP controls early acquisition of notochord phenotypic markers, collagen composition, and sonic hedgehog signaling during mouse intervertebral disc embryogenesis. Dev Biol.

[22] Bangs F, Anderson KV (2017). Primary cilia and mammalian hedgehog signaling. Cold Spring Harb Perspect Biol.

[23] Kang HJ, Yoo EJ, Lee HH et al. TonEBP promotes β-Cell survival under ER stress by enhancing Autophagy. Cells. 2020;9(9):1928. 10.3390/cells9091928. 10.3390/cells9091928 PMC7563687 32825390

[24] Herbelet S, De Vlieghere E, Gonçalves A et al. Localization and expression of Nuclear factor of activated T-Cells 5 in myoblasts exposed to pro-inflammatory cytokines or hyperosmolar stress and in biopsies from Myositis patients. Front Physiol. 2018;9:126. 10.3389/fphys.2018.00126. 10.3389/fphys.2018.00126 PMC5826317 29515464

[25] Wang G, Chen Q, Zhang X et al. PCM1 recruits Plk1 to the pericentriolar matrix to promote primary cilia disassembly before mitotic entry. J Cell Sci. 2013;126(Pt 6):1355–65. 10.1242/jcs.114918. 10.1242/jcs.114918 23345402

[26] Pugacheva EN, Jablonski SA, Hartman TR, Henske EP, Golemis EA. HEF1-dependent Aurora A activation induces disassembly of the primary cilium. Cell. 2007;129(7):1351–63. 10.1016/j.cell.2007.04.035. 10.1016/j.cell.2007.04.035 PMC2504417 17604723

[27] Ran J, Yang Y, Li D, Liu M, Zhou J. Deacetylation of α-tubulin and cortactin is required for HDAC6 to trigger ciliary disassembly. Sci Rep. 2015;5:12917. 10.1038/srep12917. 10.1038/srep12917 PMC4526867 26246421

[28] Westlake CJ, Baye LM, Nachury MV et al. Primary cilia membrane assembly is initiated by Rab11 and transport protein particle II (TRAPPII) complex-dependent trafficking of Rabin8 to the centrosome. Proc Natl Acad Sci U S A. 2011;108(7):2759–64. 10.1073/pnas.1018823108. 10.1073/pnas.1018823108 PMC3041065 21273506

[29] Knödler A, Feng S, Zhang J et al. Coordination of Rab8 and Rab11 in primary ciliogenesis. Proc Natl Acad Sci U S A. 2010;107(14):6346–51. 10.1073/pnas.1002401107. 10.1073/pnas.1002401107 PMC2851980 20308558

[30] Lu Q, Insinna C, Ott C et al. Early steps in primary cilium assembly require EHD1/EHD3-dependent ciliary vesicle formation [published correction appears in Nat Cell Biol. 2015;17(4):531]. Nat Cell Biol. 2015;17(3):228 – 40. 10.1038/ncb3109. 10.1038/ncb3109 PMC4344897 25686250

[31] Spektor A, Tsang WY, Khoo D, Dynlacht BD. Cep97 and CP110 suppress a cilia assembly program. Cell. 2007;130(4):678–90. 10.1016/j.cell.2007.06.027. 10.1016/j.cell.2007.06.027 17719545

[32] Xie S, Naslavsky N, Caplan S. EHD1 promotes CP110 ubiquitination by centriolar satellite delivery of HERC2 to the mother centriole. EMBO Rep. 2023;24(6):e56317. 10.15252/embr.202256317. 10.15252/embr.202256317 PMC10240189 37074924

[33] Lee SH, Lee MS, Choi TI et al. MCRS1 associates with cytoplasmic dynein and mediates pericentrosomal material recruitment. Sci Rep. 2016;6:27284. 10.1038/srep27284. 10.1038/srep27284 PMC4893664 27263857

[34] Inoko A, Matsuyama M, Goto H et al. Trichoplein and Aurora a block aberrant primary cilia assembly in proliferating cells. J Cell Biol. 2012;197(3):391–405. 10.1083/jcb.201106101. 10.1083/jcb.201106101 PMC3341160 22529102

[35] Arslanhan MD, Rauniyar N, Yates JR 3rd, Firat-Karalar EN. Aurora kinase a proximity map reveals centriolar satellites as regulators of its ciliary function. EMBO Rep. 2021;22(8):e51902. 10.15252/embr.202051902. 10.15252/embr.202051902 PMC8339716 34169630

[36] Choi SY, Lee-Kwon W, Kwon HM. The evolving role of TonEBP as an immunometabolic stress protein. Nat Rev Nephrol. 2020;16(6):352–64. 10.1038/s41581-020-0261-1. 10.1038/s41581-020-0261-1 32157251

[37] Jauliac S, López-Rodriguez C, Shaw LM, Brown LF, Rao A, Toker A. The role of NFAT transcription factors in integrin-mediated carcinoma invasion. Nat Cell Biol. 2002;4(7):540–4. 10.1038/ncb816. 10.1038/ncb816 12080349

[38] Pearson CG, Culver BP, Winey M. Centrioles want to move out and make cilia. Dev Cell. 2007;13(3):319–21. 10.1016/j.devcel.2007.08.007. 10.1016/j.devcel.2007.08.007 17765674

[39] Wu CT, Chen HY, Tang TK. Myosin-Va is required for preciliary vesicle transportation to the mother centriole during ciliogenesis. Nat Cell Biol. 2018;20(2):175–85. 10.1038/s41556-017-0018-7. 10.1038/s41556-017-0018-7 29335527

[40] Lee SH, Joo K, Jung EJ, Hong H, Seo J, Kim J (2018). Export of membrane proteins from the golgi complex to the primary cilium requires the kinesin motor, KIFC1. FASEB J.

[41] Dammermann A, Merdes A. Assembly of centrosomal proteins and microtubule organization depends on PCM-1. J Cell Biol. 2002;159(2):255–66. 10.1083/jcb.200204023. 10.1083/jcb.200204023 PMC2173044 12403812

[42] Chamling X, Seo S, Searby CC, Kim G, Slusarski DC, Sheffield VC. The centriolar satellite protein AZI1 interacts with BBS4 and regulates ciliary trafficking of the BBSome. PLoS Genet. 2014;10(2):e1004083. 10.1371/journal.pgen.1004083. 10.1371/journal.pgen.1004083 PMC3923683 24550735

[43] Hall EA, Keighren M, Ford MJ et al. Acute versus chronic loss of mammalian Azi1/Cep131 results in distinct ciliary phenotypes. PLoS Genet. 2013;9(12):e1003928. 10.1371/journal.pgen.1003928. 10.1371/journal.pgen.1003928 PMC3887133 24415959

[44] Kurtulmus B, Yuan C, Schuy J et al. LRRC45 contributes to early steps of axoneme extension. *J Cell Sci*. 2018;131(18):jcs223594. Published 2018 Sep 20. 10.1242/jcs.223594. 10.1242/jcs.223594 30131441

[45] Kamiya A, Tan PL, Kubo K (2008). Recruitment of PCM1 to the centrosome by the cooperative action of DISC1 and BBS4: a candidate for psychiatric illnesses. Arch Gen Psychiatry.

