# Supplementary material for: TonEBP inhibits ciliogenesis by controlling aurora kinase A and regulating centriolar satellite integrity
Source: Cell Commun Signal. 2024 Jul 3;22:348. doi: 10.1186/s12964-024-01721-8 (PMC11221002; doi:10.1186/s12964-024-01721-8)
Supplement: Supplementary file 1 — Supplementary Material 1 [file 12964_2024_1721_MOESM1_ESM.docx]

**­­­­­­­­­Additional file:**

**TonEBP inhibits ciliogenesis by controlling aurora kinase A and regulating centriolar satellite integrity**

**Batchingis Chinbold^1^, Hyug Moo Kwon^2^ and Raekil Park^1*^**

^1^ Department of Biomedical Science and Engineering, Gwangju Institute of Science and Technology, Gwangju 61005, Republic of Korea

^2^ School of Life Sciences, Ulsan National Institute of Science and Technology, Ulsan, Republic of Korea

***Correspondence:**

# Raekil Park, M.D., Ph.D.

# Department of Biomedical Science & Engineering,

# Gwangju Institute of Science and Technology, Gwangju 61005, Republic of Korea

# Tel.: +82-62-715-5361; Fax: +82-62-715-5309; E-mail: rkpark@gist.ac.kr

# **
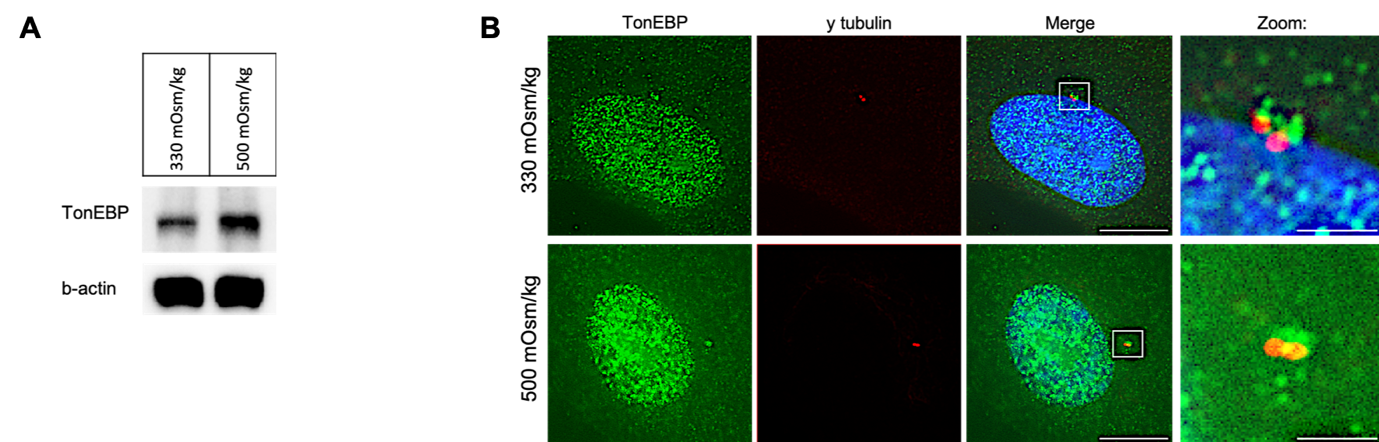
**

**Supplementary Figure 1. Pericentriolar localization of TonEBP is not dependent on tonicity.**

1. RPE1 cells were treated with hypertonic medium for 24 h and subjected to Western blot.
2. Cells were treated with hypertonic medium for 24 h and immunostained for TonEBP (green) and γ-tubulin (red). Scale bar, 10um. Zoom scale bar, 2 µm.


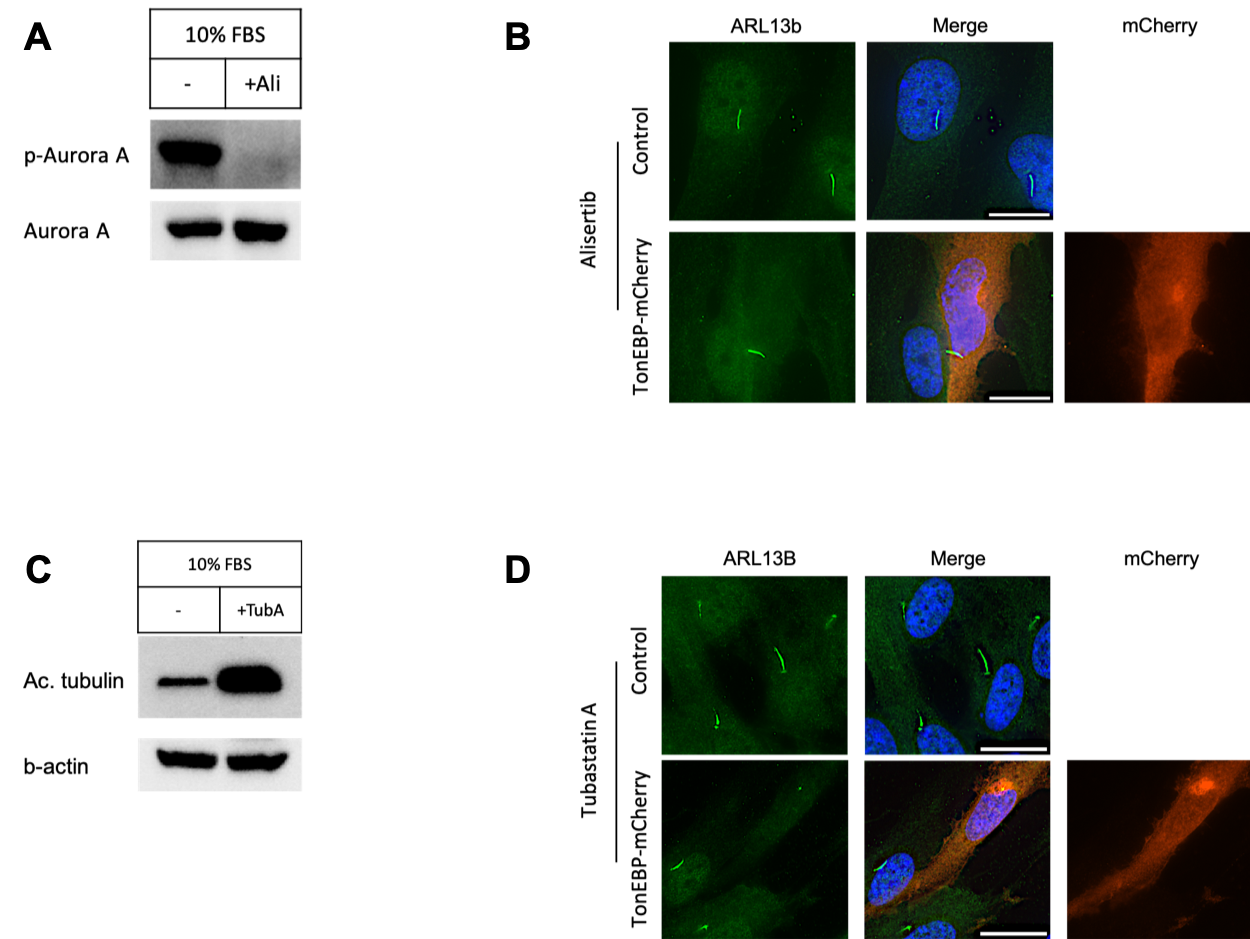


**Supplementary Figure 2: Aurora kinase A - HDAC6 pathway is not the primary mediator of TonEBP regulated ciliogenesis.**

1. RPE1 cells were treated with Alisertib (5 µM) for 24 h and subjected to Western blot.
2. Cells were transfected with TonEBP-mCherry for 24 h, followed by Alisertib (5 uM) treatment for 24 h, and immunostained for ARL13b (green) and mCherry (red). Scale bar, 20 µm
3. Cells were treated with Tubastatin A (5 µM) for 24 h and subjected to Western blot.
4. Cells were transfected with TonEBP-mCherry for 24 h, followed by Tubastatin A (5 uM) treatment for 24 h, and immunostained for ARL13b (green) and mCherry (red). Scale bar, 20 µm.


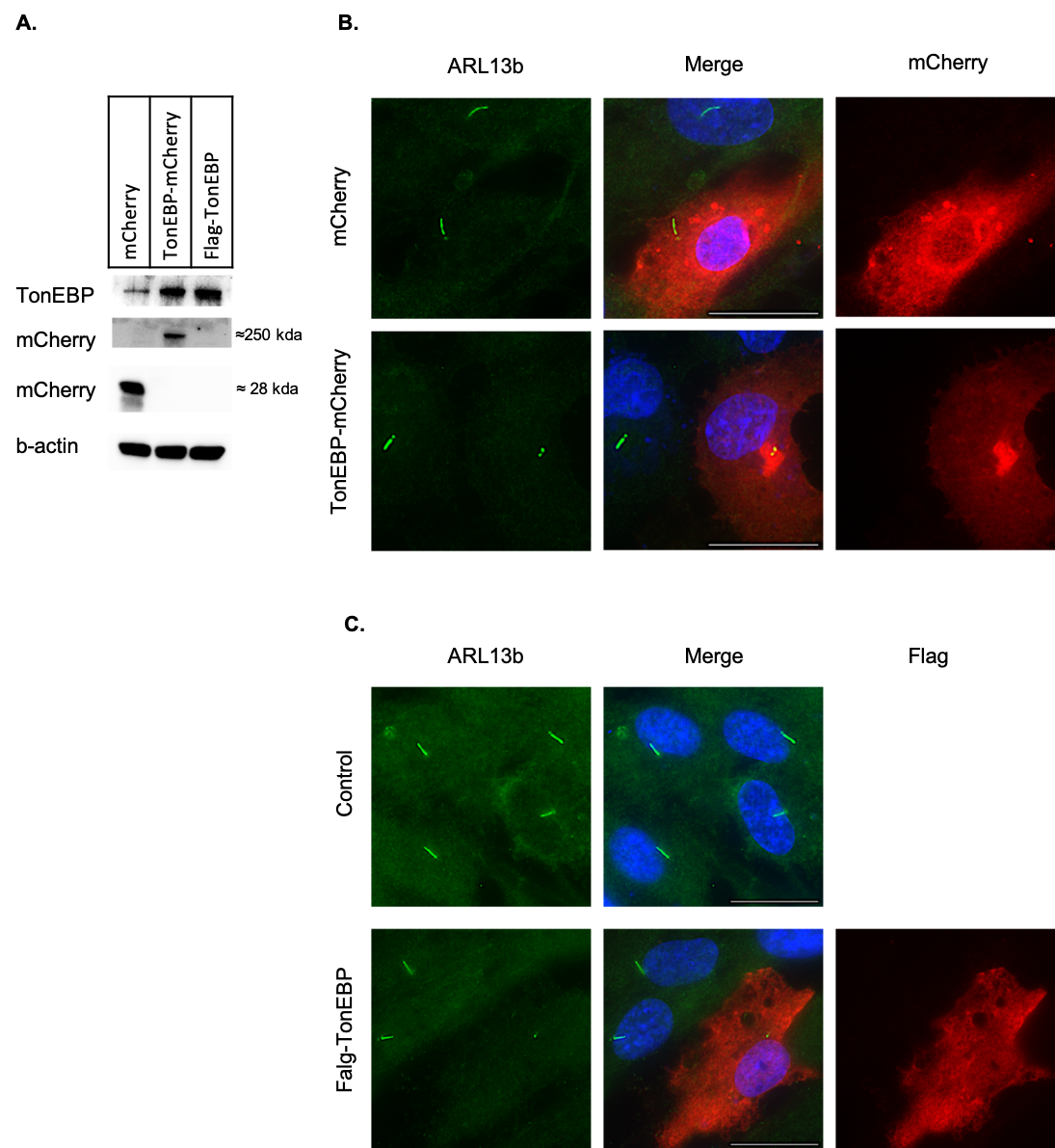


**Supplementary Figure 3: TonEBP overexpression inhibits ciliogenesis**

1. RPE1 cells were transfected with mCherry, TonEBP-mCherry, Flag-TonEBP for 24 h, followed by serum starvation for additional 24 h. Cells were harvested and subjected to Western blot.
2. Cells were transfected with mCherry and TonEBP-mCherry for 24 h, followed by serum starvation for 24 h, and immunostained for ARL13b (green) and mCherry (red). Scale bar, 40 µm.
3. Cells were transfected with Flag-TonEBP for 24 h, followed by serum starvation for 24 h, and immunostained for ARL13b (green) and Flag (red). Scale bar, 40 µm.


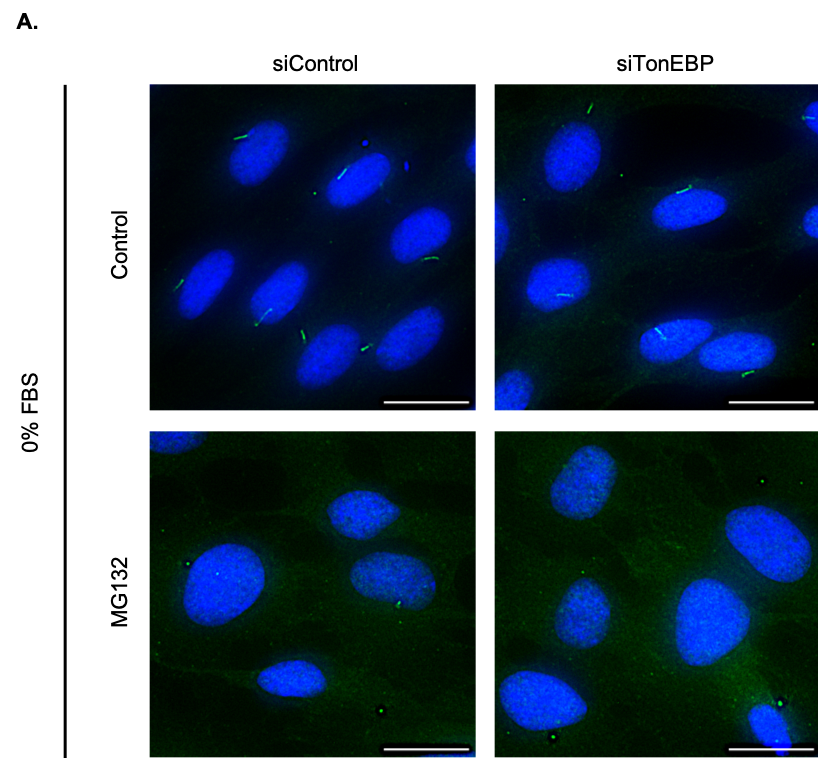


**Supplementary Figure 4: MG132 inhibits ciliogenesis**

1. RPE1 cells were transfected with TonEBP siRNA for 24 h, followed by serum starvation with MG132 (5uM) for 24 h. Cells were harvested and subjected to Immunostained with ARL13b (green) and Dapi (blue.) Scale bar, 40 µm.


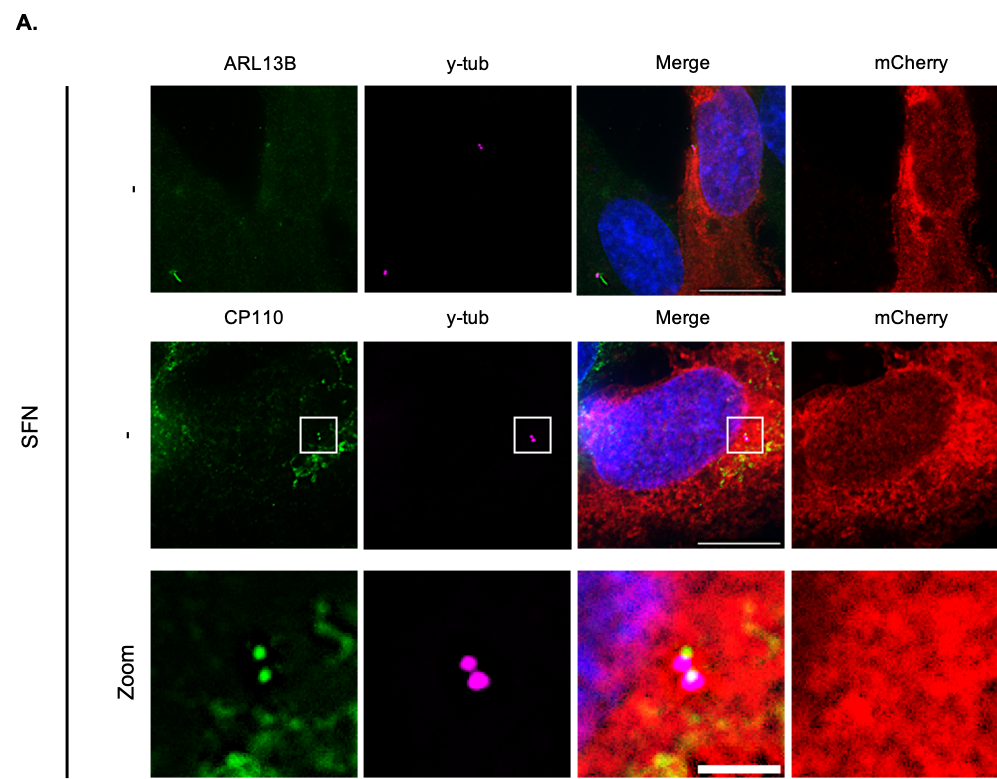


**Supplementary Figure 5: Surlforaphane failed to rescue ciliogenesis**

1. RPE1 cells were transfected with TonEBP-mCherry plasmid for 24 h, followed by serum starvation with Sulforapahne (10 uM) for 24 h. Cells were harvested and subjected to Immunostained with ARL13b (green) or CP110 (green), y-tubulin (magenta), mCherry (red) and Dapi (blue). Scale bar, 40 µm. Zoom scale bar, 2um


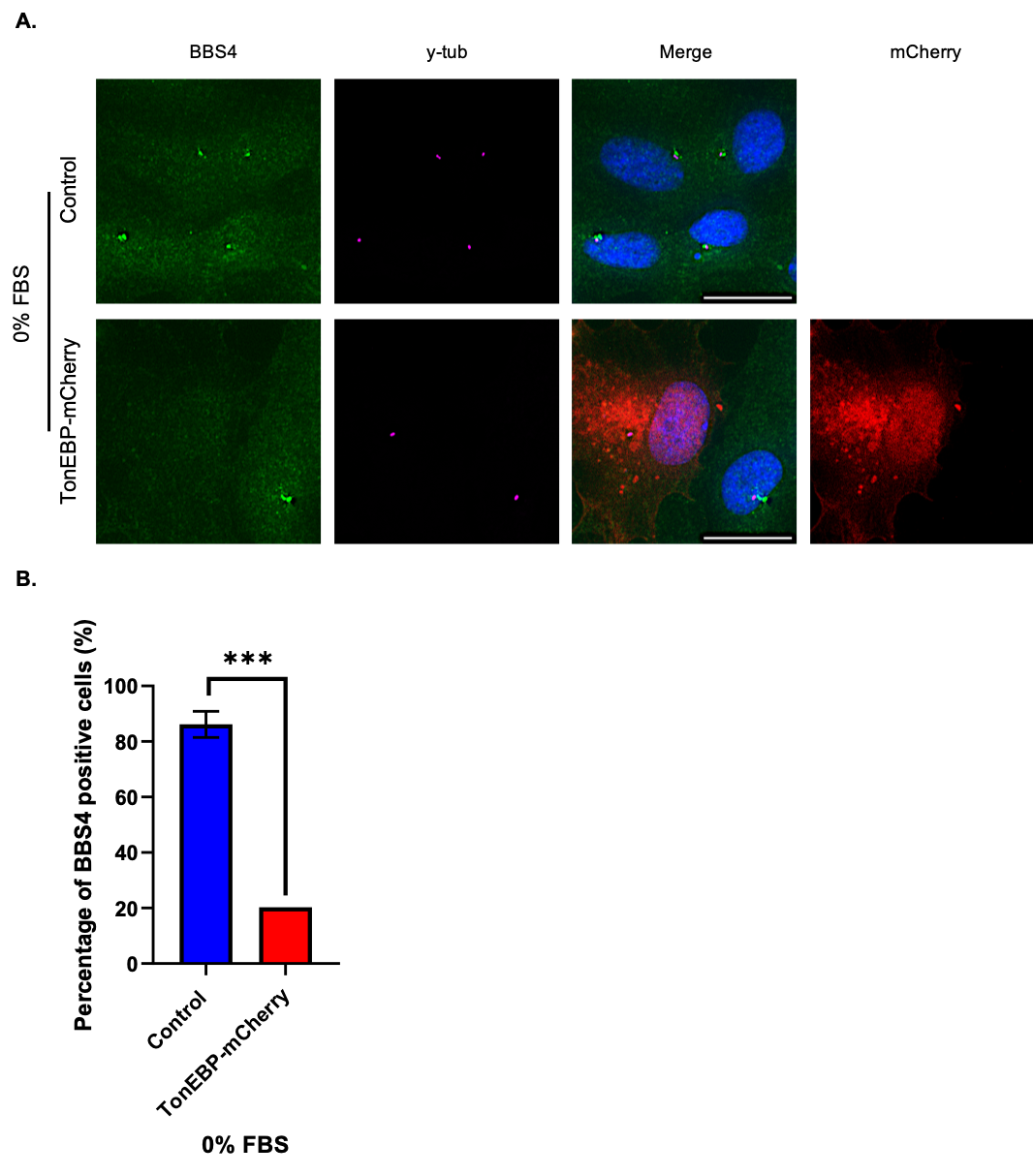


**Supplementary Figure 6: TonEBP overexpression regulates BBS4 localization**

1. RPE1 cells were transfected with TonEBP-mCherry plasmid for 24 h, followed by serum starvation for 24 h. Cells were harvested and subjected to Immunostained with BBS4 (green), y-tubulin (magenta), mCherry (red) and Dapi (blue). Scale bar, 40 µm.
2. Quantification of BBS4 positive cells as shown in (A). Data is represented as mean ± SD (n = 3 experiments). Fifty cells were scored per condition per experiment; ***P<0.001, Student’s t-test.
