# Supplementary material for: TonEBP inhibits ciliogenesis by controlling aurora kinase A and regulating centriolar satellite integrity
Source: Cell Commun Signal. 2024 Jul 3;22:348. doi: 10.1186/s12964-024-01721-8 (PMC11221002; doi:10.1186/s12964-024-01721-8)
Supplement: Supplementary file 2 — Supplementary Material 2 [file 12964_2024_1721_MOESM2_ESM.docx]

**­­­­­­­­­Supplementary file:**

**TonEBP inhibits ciliogenesis by controlling aurora kinase A and regulating centriolar satellite integrity**

**Batchingis Chinbold^1^, Hyug Moo Kwon^2^ and Raekil Park^1*^**

^1^ Department of Biomedical Science and Engineering, Gwangju Institute of Science and Technology, Gwangju 61005, Republic of Korea

^2^ School of Life Sciences, Ulsan National Institute of Science and Technology, Ulsan, Republic of Korea

***Correspondence:**

# Raekil Park, M.D., Ph.D.

# Department of Biomedical Science & Engineering,

# Gwangju Institute of Science and Technology, Gwangju 61005, Republic of Korea

# Tel.: +82-62-715-5361; Fax: +82-62-715-5309; E-mail: rkpark@gist.ac.kr

**Figure 2.**

**
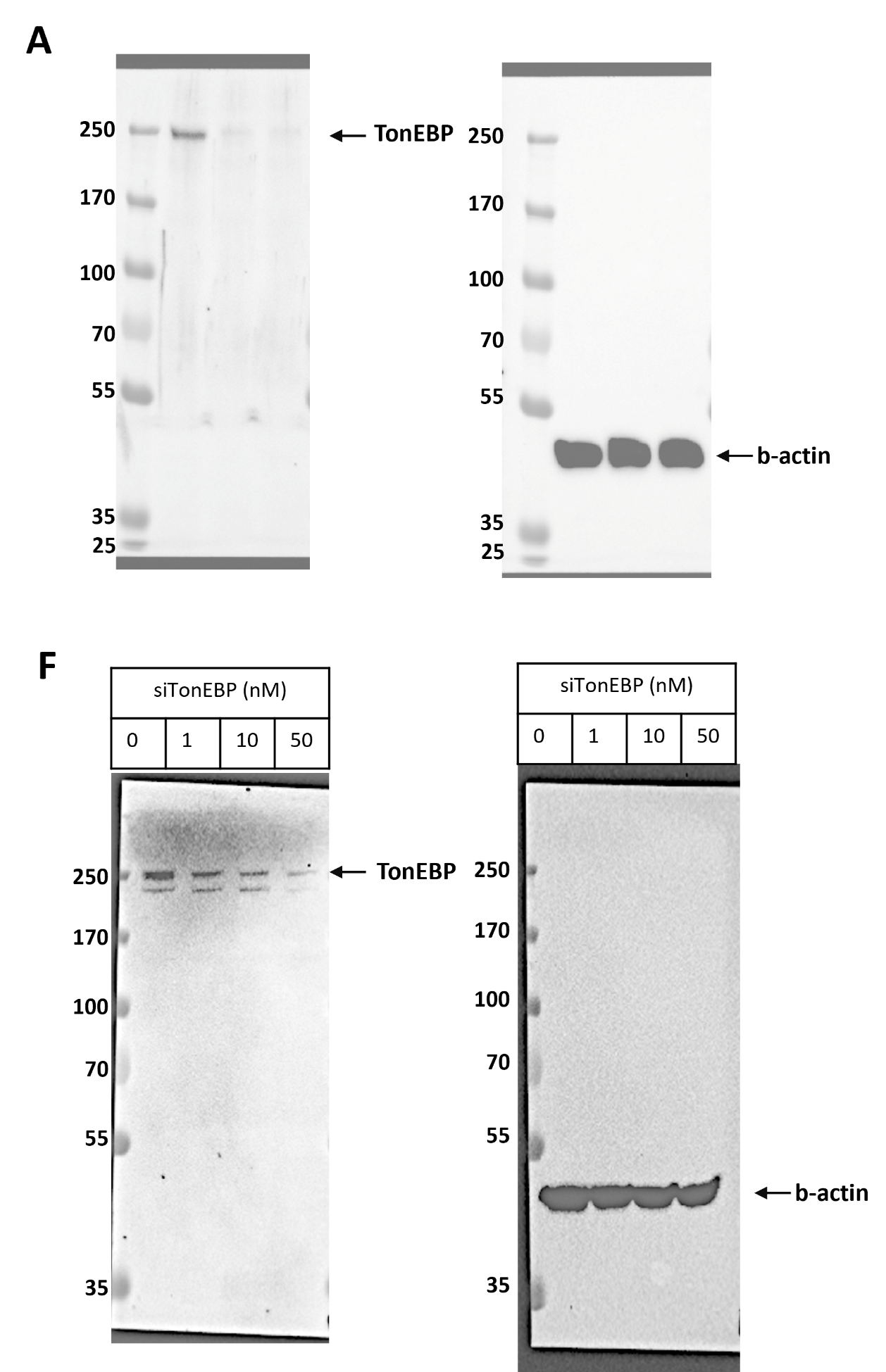
Figure 3.**

**
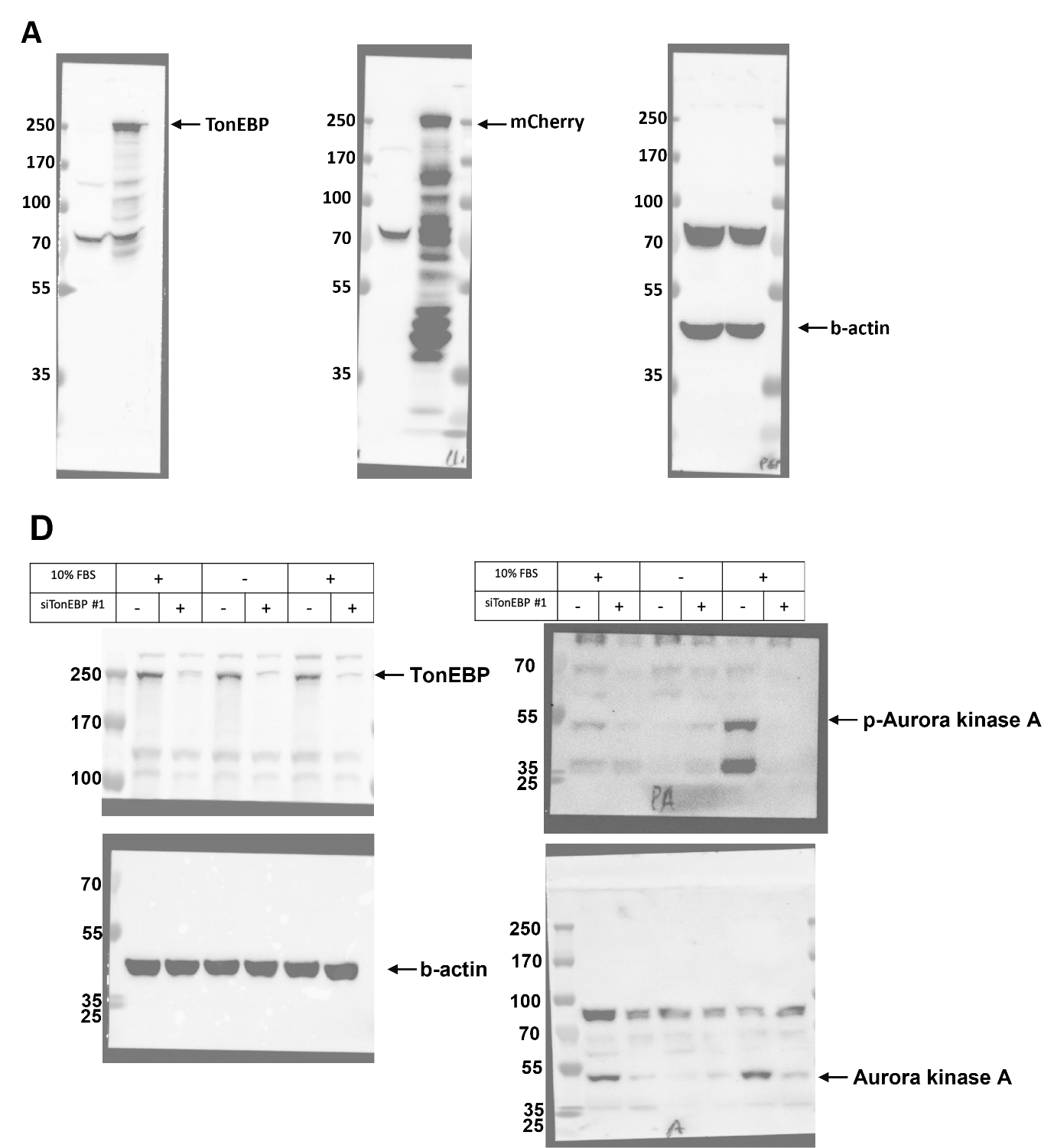
**

**Figure 4.**

**
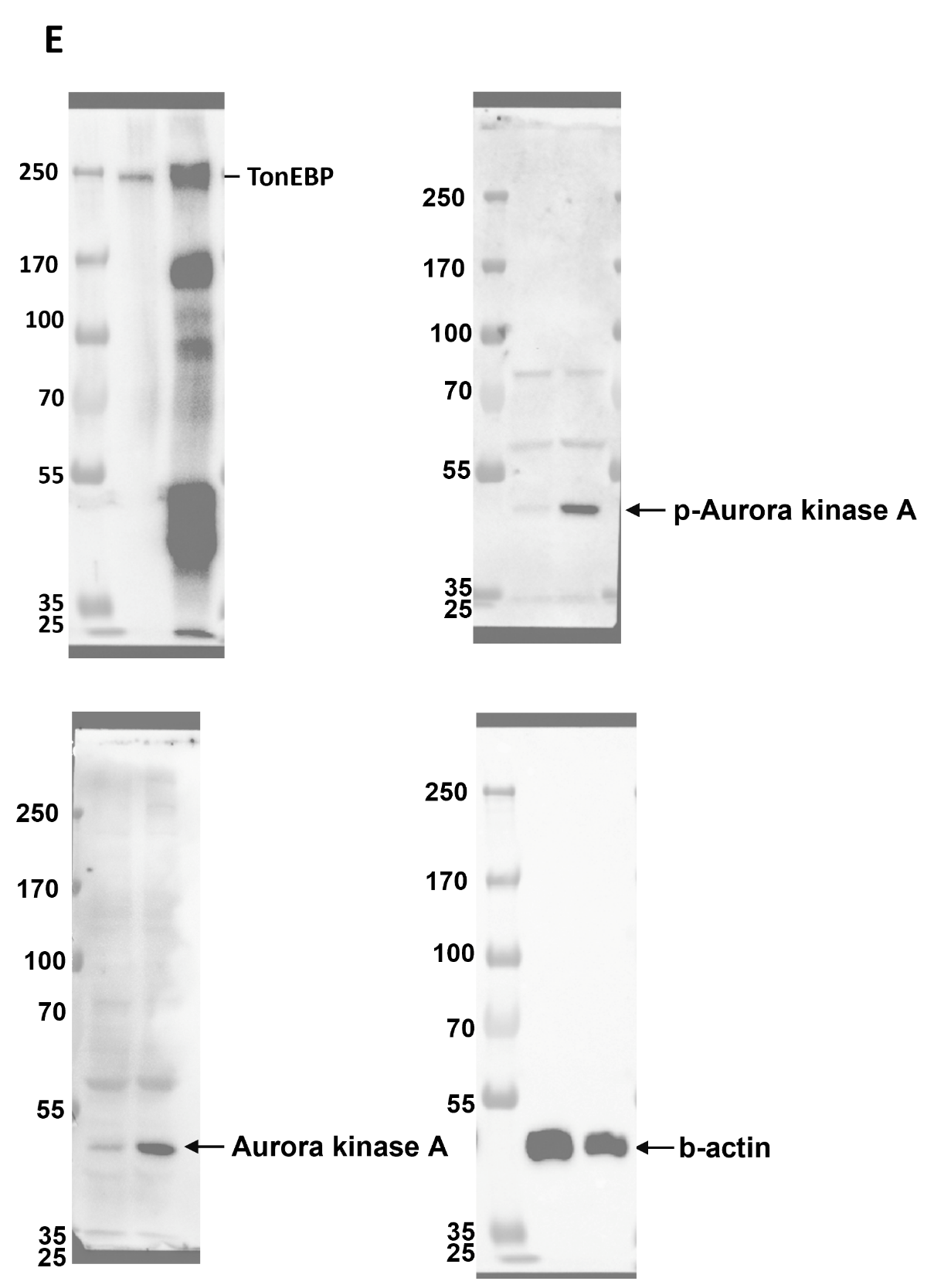
**

**Supplementary Figure 1.**

**
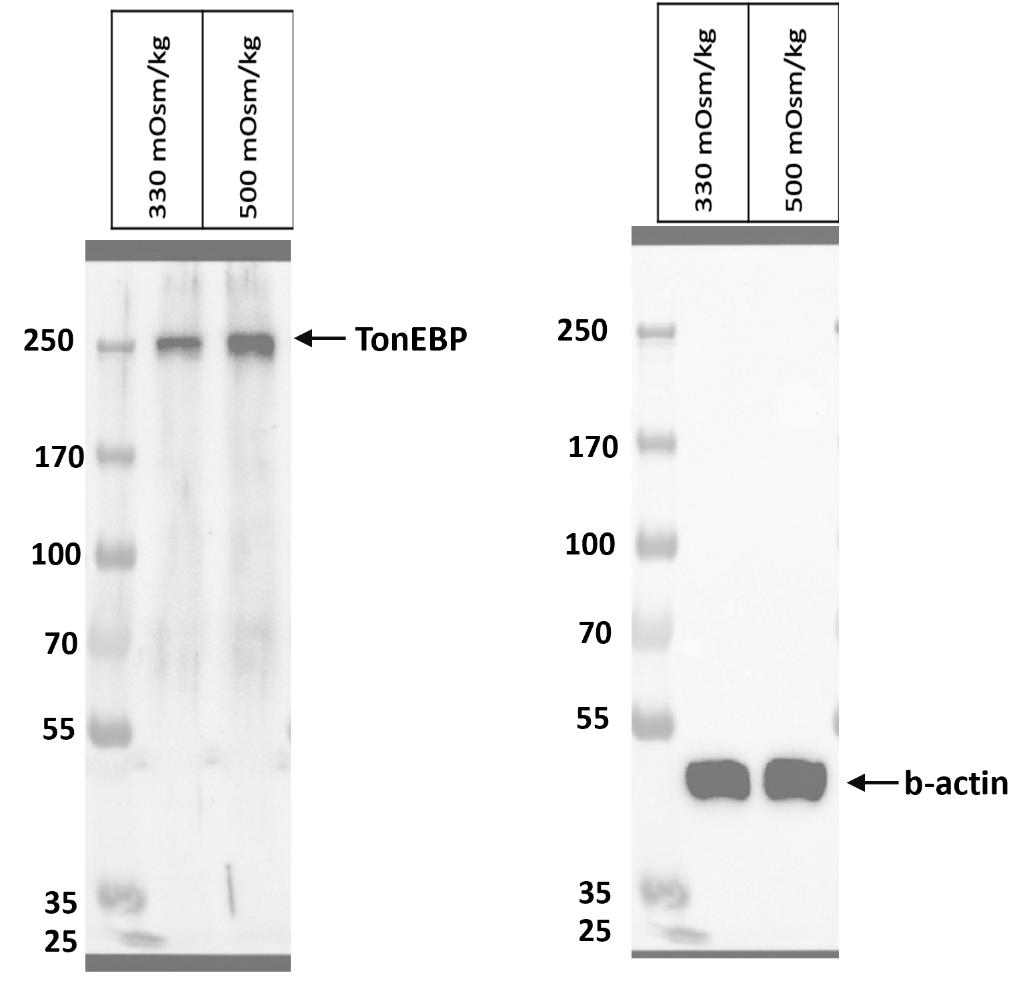
**

**Supplementary Figure 2**.


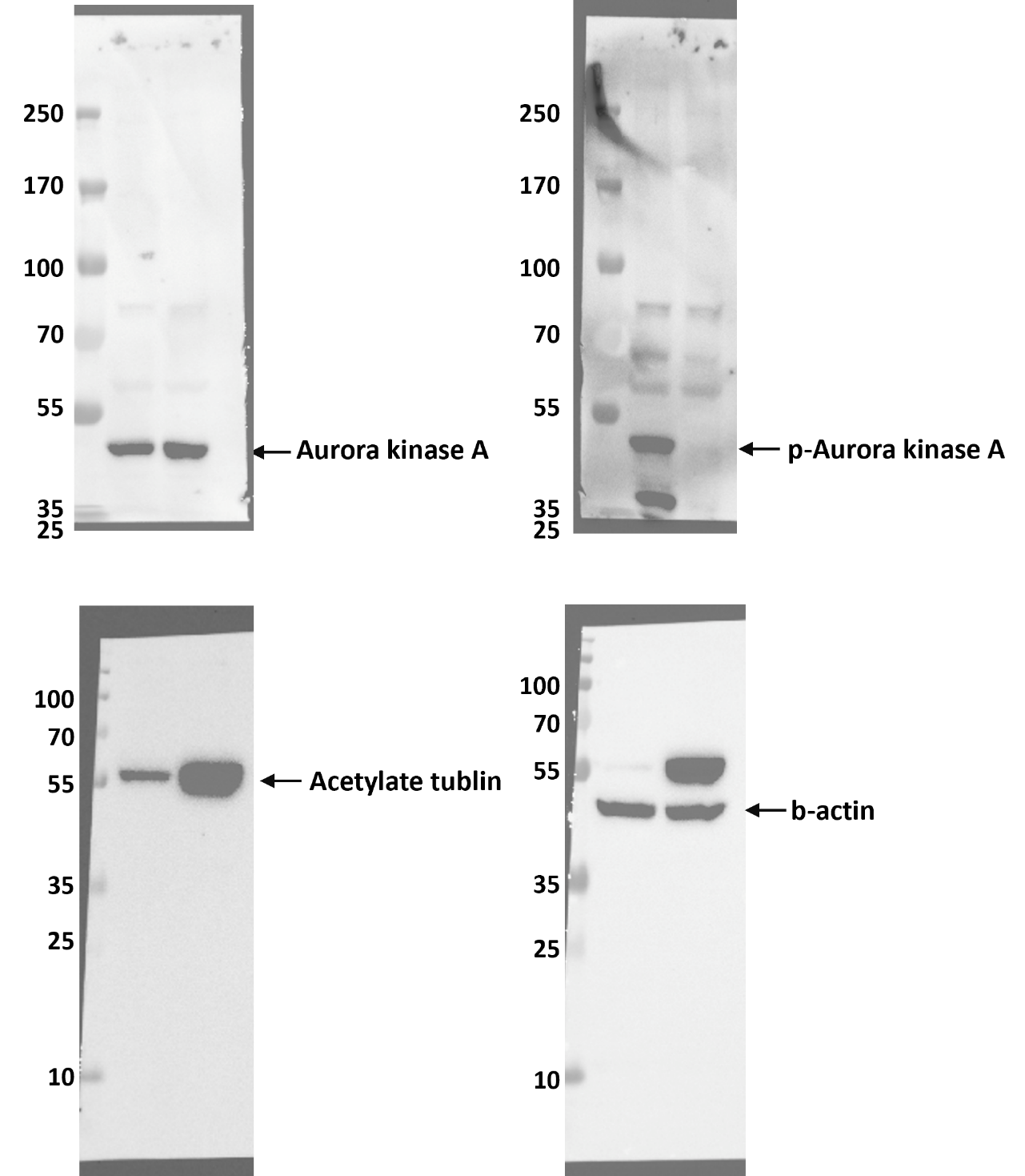


**Supplementary Figure 3**.

**
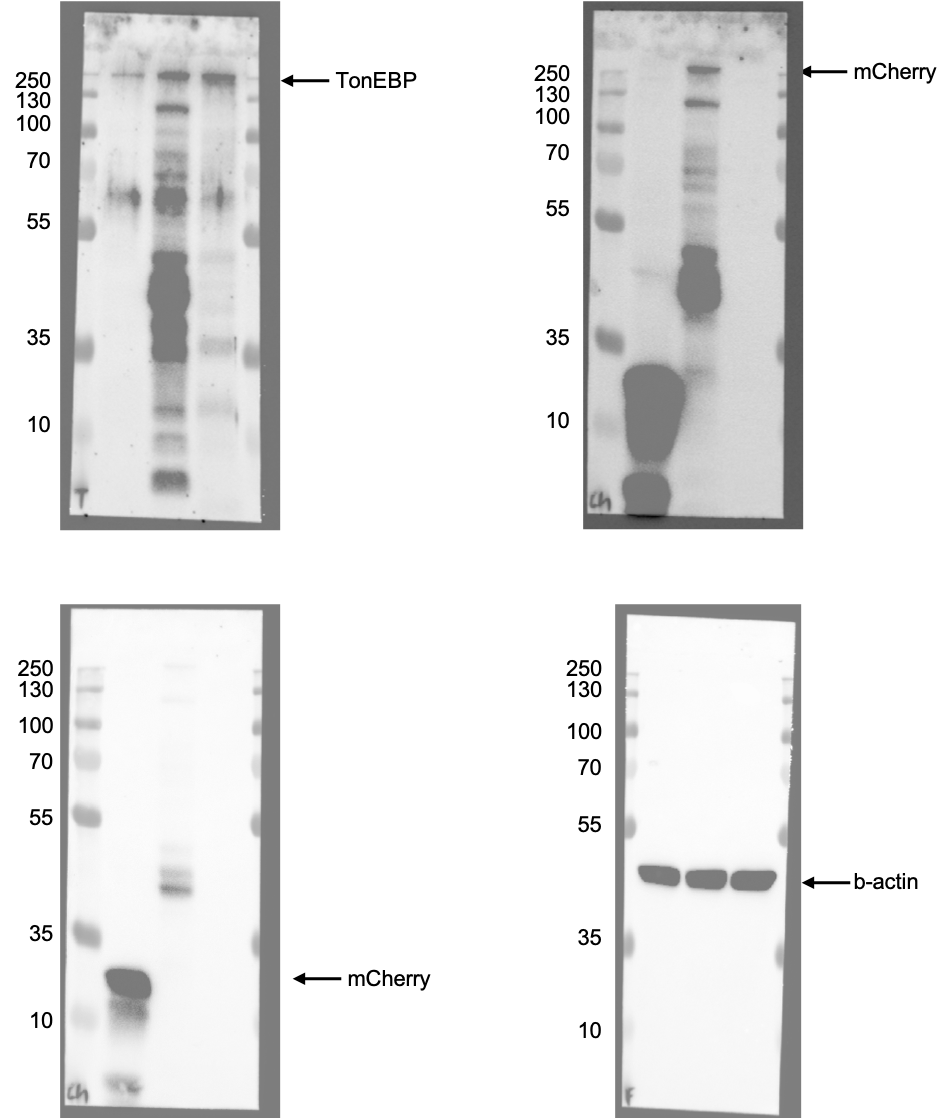
**
